# STING COPII ER Export Trafficking and Signaling Primed by Phosphorylation Switches

**DOI:** 10.1002/advs.202503660

**Published:** 2025-07-01

**Authors:** Yanan Nan, Dongxiao Cui, Jiajian Guo, Xiaojing Ma, Jiaming Wang, Linyue Guo, Tianyu Li, Mingrui Yang, Guangrui Huang, Anlong Xu, Wenfu Ma

**Affiliations:** ^1^ School of Life Sciences Beijing University of Chinese Medicine Beijing 102488 China; ^2^ Key Laboratory of Shaanxi Administration of Traditional Chinese Medicine for TCM Compatibility Shaanxi University of Chinese Medicine Xianyang 712046 China

**Keywords:** STING, TBK1, IRF3, COPII vesicle trafficking, ER export, interferon, phosphorylated motifs

## Abstract

Despite advances in understanding the STING signaling pathway, mechanisms governing cyclic GMP‐AMP (cGAMP)‐induced STING trafficking out of the endoplasmic reticulum (ER) remain unclear. This study reveals that STING localization is regulated by the balance between coat protein II (COPII)‐ and coat protein I (COPI)‐mediated trafficking, maintaining ER residency in the inactive state or promoting transport to the *cis*‐Golgi via enhanced COPII‐mediated export upon activation. Two novel TANK‐binding kinase 1 (TBK1)‐regulated phosphorylated COPII sorting signals on STING—a conserved pSGME motif and a primate‐specific pFS motif—are biochemically and structurally identified. These cGAMP‐induced signals drive activated STING toward the ER‐Golgi intermediate compartment (ERGIC) and the *cis*‐Golgi complex. Using a cell‐free COPII vesicle reconstitution system, TBK1 activation is shown to occur on COPII vesicles, while IRF3 phosphorylation is confined to the ERGIC or the *cis*‐Golgi complex post‐uncoating, due to the competitive binding of COPII Sec24 and IRF3 to phosphorylated STING. A class of compounds is also identified that attenuates IRF3 phosphorylation by inhibiting phosphorylated STING packaging into COPII vesicles. These findings elucidate STING trafficking mechanisms and offer therapeutic potential for diseases linked to dysregulated STING activation.

## Graphical abstract

This schematic outlines STING trafficking and signaling via coat protein II (COPII)‐mediated endoplasmic reticulum (ER) export. Following cyclic GMP‐AMP (cGAMP) stimulation, TANK‐binding kinase 1 (TBK1) activates on COPII vesicles, partially dissociating into the cytosol to phosphorylate ER‐localized STING, creating pSGME/pFS COPII‐sorting motifs. Competition at shared phosphorylation sites limitsinterferon regulatory factor 3 (IRF3) activation to post‐uncoating ER ‐ Golgi intermediate compartment (ERGIC) or cis‐Golgi compartments, emphasizing spatially compartmentalized signaling.

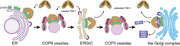

## 1 Introduction

The cyclic GMP‐AMP synthase‐stimulator of interferon genes (cGAS‐STING) pathway plays a pivotal role in host defense against microbial infections, immune surveillance, and the regulation of inflammatory diseases, positioning it as one of the central focuses in immunological and therapeutic research.^[^ ^1^, ^2^, ^3^, ^4^ ^]^ Dysregulated activation of this pathway is implicated in the pathogenesis of autoimmune diseases, including Aicardi‐Goutières syndrome (AGS),^[^ ^5^ ^]^ STING‐associated vasculopathy with onset in infancy (SAVI),^[^ ^6^ ^]^ systemic lupus erythematosus (SLE),^[^ ^7^, ^8^ ^]^ and chronic inflammatory disorders.^[^ ^9^, ^10^ ^]^ Upon detecting cytosolic double‐stranded DNA (dsDNA) from invading pathogens, cellular damage, or mitochondrial stress, the DNA sensor cGAS synthesizes the second messenger cyclic GMP‐AMP (cGAMP).^[^ ^11^, ^12^ ^]^ This molecule binds to and activates STING, an endoplasmic reticulum (ER) transmembrane protein. Following activation, STING is packaged into coat protein II (COPII) vesicles and transported to ER‐Golgi intermediate compartment (ERGIC) and then the Golgi apparatus, where it recruits and activates TANK‐binding kinase 1 (TBK1). This cascade leads to the phosphorylation and activation of interferon regulatory factor 3 (IRF3), which translocates to the nucleus to induce the transcription of type I interferons (IFNs) and pro‐inflammatory cytokines, thereby orchestrating innate immune responses.^[^ ^13^, ^14^ ^]^

In the early secretory pathway, COPII vesicles mediate anterograde transport, shuttling cargo from the ER to the Golgi complex, coat protein I (COPI) vesicles facilitate retrograde transport, retrieving ER‐resident proteins from the ERGIC or the Golgi complex back to the ER.^[^ ^15^, ^16^ ^]^ The sorting of secretory proteins into COPII or COPI vesicles is typically governed by specific trafficking signals. For instance, in mammals, motifs such as the LxxLE, DxE, and ΦC are recognized by the B site of Sec24a or Sec24b of COPII coat,^[^ ^17^, ^18^ ^]^ whereas the IxM motif is recognized by Sec24c or Sec24d.^[^ ^19^ ^]^ Similarly, the KxKxx or KKxx dilysine motifs are recognized by the α‐COP or β'‐COP subunits of the COPI coat.^[^ ^19^, ^20^ ^]^ The intricate balance between COPII‐ and COPI‐mediated trafficking ensures the precise cellular localization of secretory cargo. In the case of STING, the Surf4 protein, which functions as a COPI vesicle trafficking adapter, has been reported to mediate STING retrieval trafficking. The binding of cGAMP disrupts STING and Surf4 association, leading to the failure of STING retrieval trafficking and thus allowing STING trafficking forward.^[^ ^21^ ^]^

Although much of the cGAS‐STING signaling pathway has been elucidated, a critical question remains unresolved: how is STING transported out of the ER via COPII vesicle trafficking, and why is signaling so tightly coupled to STING's ER export upon activation by cGAMP?^[^ ^9^, ^14^, ^22^, ^23^ ^]^ In contrast to its ER export, the Golgi‐to‐lysosome transport of STING is well characterized, involving its packaging into clathrin‐coated AP‐1 vesicles via a phosphorylation‐enhanced dileucine motif, ultimately leading to STING degradation in the lysosome.^[^ ^24^ ^]^ Despite the established importance of STING ER export in signal transduction,^[^ ^25^, ^26^, ^27^, ^28^ ^]^ the molecular mechanisms governing this process remain enigmatic. To elucidate the unresolved question, in this study, we first demonstrated that STING's ER residence was regulated by the balance between COPI and COPII trafficking. Subsequently, we biochemically and structurally identified two TBK1‐phosphorylated motifs in STING including pSGME and pFS as authentic COPII sorting signals. We then showed the mechanistic explanation for how cGAMP binding disrupted STING's trafficking equilibrium by generating these novel COPII sorting signals, ultimately promoting its export from the ER. Importantly, we further delineated the sequential and spatial activation of downstream STING signaling, revealing that TBK1 was activated by cGAMP‐bound STING on COPII vesicles or cellular organelles, whereas IRF3 phosphorylation occurred exclusively at the organelles following COPII vesicle uncoating. Additionally, we discovered a class of compounds that competitively bind to COPII in a manner analogous to STING, thereby inhibiting STING activation. Our findings not only unraveled the long‐standing enigma of STING trafficking through COPII vesicles but also offered a promising therapeutic approach for mitigating autoimmune diseases driven by STING overactivation.

## 2 Results

### 2.1 COPI‐COPII Trafficking Dynamics Shapes STING Early Secretory Organelle Localization

**Figure 1:**
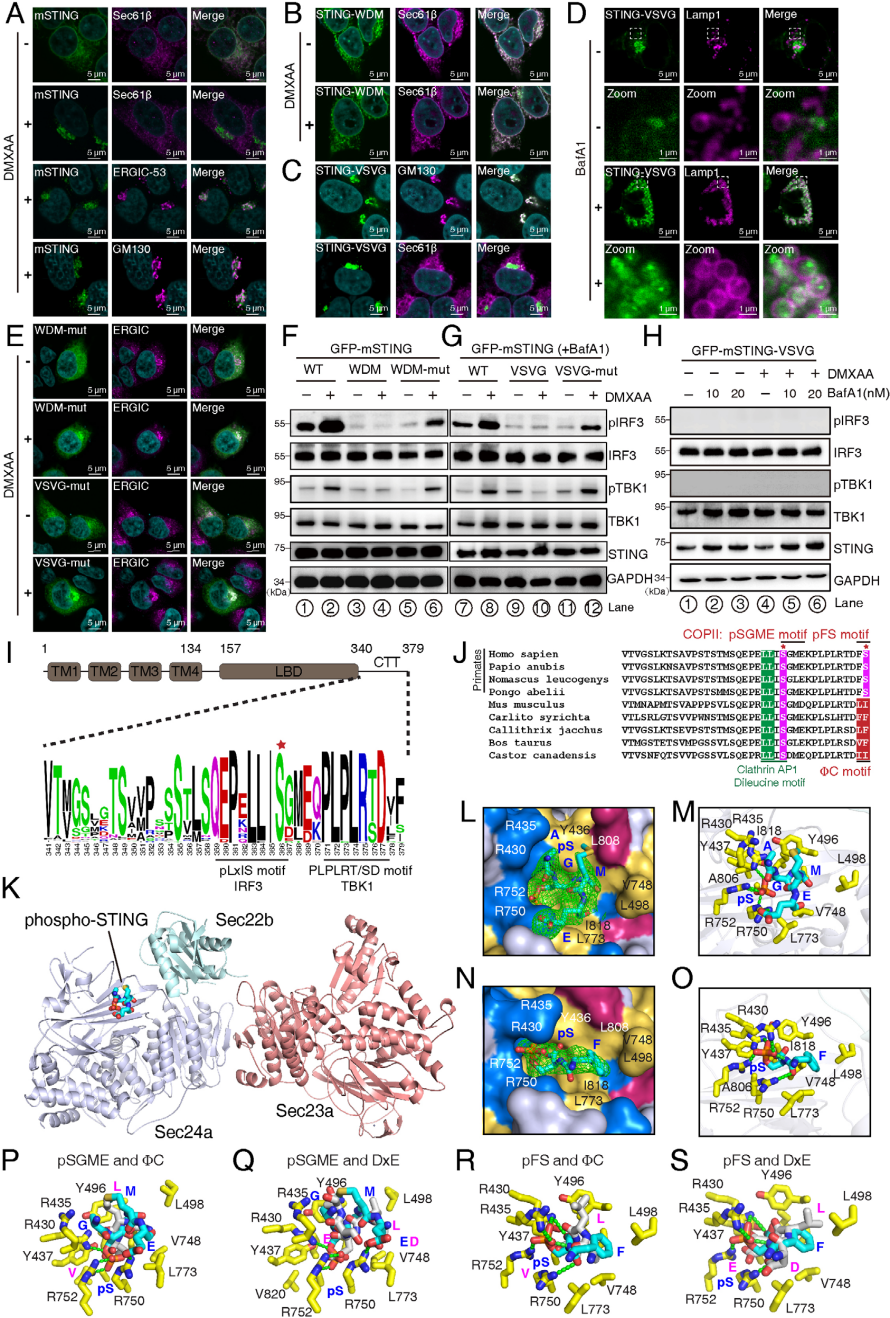
Structural characterization of STING pSGME and pFS motifs bound to COPII Sec24a. A) Subcellular localization of mouse STING following treatment with 100 µM DMXAA for 45 min. The ER is labeled with Sec61β (purple), the ERGIC with ERGIC‐53 (purple), and the *cis*‐Golgi complex with GM130 (purple). STING is shown in green. B) Colocalization of STING‐WDM with the ER marker Sec61β after 45 min of DMXAA treatment, demonstrating ER retention. C) Trafficking of STING‐VSVG to the *cis*‐Golgi complex in the absence of activation. D) STING‐VSVG accumulation in lysosomes treated by BafA1. E–G) Mutations of trafficking signals in WDM dilysine motif (WDM: AKEKSD; WDM‐mut: AAEASD) or DxE motif in VSVG (VSVG: QIYTDIEMNRLGK; VSVG‐mut: QIYTAIAMNRLGK) restores STING trafficking (E) or downstream activation (F, G). Western blot analysis showing activation of WT STING, but not STING‐WDM or STING‐VSVG, following DMXAA treatment. The HEK‐293T cells in (G) were pretreated with 20 nM BafA1 for 1 h. H) Western blot analysis revealing BafA1 treatment rescued STING‐VSVG stability. I, J) Sequence alignments of the STING CTT across 190 mammalian species (I),^[^ ^44^ ^]^ with a focus on primates versus other mammals, highlighting conserved signaling regions (J). The TBK1 phosphorylated S is indicated by a red star. K–O) Structural details of the Sec23a/24a/22b complex bound to the human STING pSGME motif (PDB ID: 9UVD) (K), presented in surface representation (L) and detailed binding interactions in stick style (M), or bound to the pFS motif (PDB ID: 9UVE) in surface representation (N) and stick style (O). The phosphorylated STING motifs are depicted in cyan, with the *Fourier* difference electron density maps contoured at 2.0 σ against a resolution of 2.5 Å for (L) and 2.9 Å for (N). Key residues of the Sec24a B site are highlighted in yellow, and all interaction bonds between pSGME or pFS with B site are indicated by green dashed lines. P–S) The comparison of the pSGME motif with the ΦC motif (PDB ID: 5VNE) (P) and the DxE motif (PDB ID: 1PD1) (Q), as well as the pFS motif with the ΦC (R) and the DxE motif (S). The key residues of the Sec24 B site are displayed as yellow sticks. The pSGME and pFS motifs are highlighted in cyan, while the DxE and ΦC motifs are depicted in gray‐white.

It is well established that cGAMP binding induces STING export from the ER, a critical step for initiating downstream signaling events, including the activation of TBK1 and phosphorylation of IRF3. To investigate this process, we employed HEK‐293T cells stably expressing mouse STING tagged with an N‐terminal green fluorescent protein (GFP), where we chose mouse STING due to its cell‐permeable agonist 5,6‐dimethylxanthenone‐4‐acetic acid (DMXAA) to simplify the experimental process.^[^ ^29^, ^30^, ^31^ ^]^ Treatment with DMXAA for 45 min, STING was observed to translocate from the ER to ERGIC or the *cis*‐Golgi complex (**Figure** **1**A). To characterize STING‐mediated signaling activation, we employed HEK‐293T cells expressing STING either transiently or stably to assess the downstream signaling in Figure 1F,G or Figure S2A,B, respectively, which displayed that STING relocalization coincided with the activation of downstream TBK1 and IRF3 signaling (Figure 1F,G, lane 1–2, 7–8, Figure S1A, lane 1–2 and S1B, lane 1–3 (Supporting Information), consistent to the documented studies about STING trafficking and activation.^[^ ^14^, ^29^, ^32^ ^]^ To further examine the necessity of STING export from the ER for effective signal transduction, we engineered a GFP‐tagged mouse STING variant fused with the WDM (WBP1 dilysine motif: AKEKSD) at its C‐terminus,^[^ ^33^ ^]^ referred to as STING‐WDM for simplicity. The WDM motif is a potent COPI sorting signal,^[^ ^33^, ^34^ ^]^ ensuring strong ER retention through COPI‐mediated retrograde trafficking. Following DMXAA stimulation, STING‐WDM exhibited predominant ER retention (Figure 1B), and the negligible activation of TBK1 and IRF3 was observed (Figure 1F, lane 3–4). The mutation of the dilysine motif in WDM restored STING ER export trafficking and downstream activation, consistent to the designed functional role of WDM motif (Figure 1B,E,F lane 3–6). These findings underscored the essential role of STING export from the ER in initiating downstream signaling.

To investigate whether STING ER export is able to be enhanced through COPII exporting signals, we fused a potent COPII‐sorting ER export motif, the VSV G tag,^[^ ^19^, ^35^ ^]^ to the C‐terminus of GFP‐tagged mouse STING, generating the construct termed STING‐VSVG. As shown in Figure 1C, the VSV G tag effectively directed STING trafficking predominantly to the *cis*‐Golgi complex. However, STING‐VSVG exhibited significantly reduced abundance compared to wild‐type (WT) STING, potentially due to lysosomal degradation (Figure S1B (Supporting Information), WT: lane 1–3 and VSVG: lane 4–6).^[^ ^24^, ^36^, ^37^ ^]^ To investigate this, we treated cells with bafilomycin A1 (BafA1), a selective inhibitor of lysosomal V‐ATPase,^[^ ^38^, ^39^ ^]^ to determine whether STING‐VSVG turnover was lysosome‐dependent. Notably, BafA1 treatment substantially enhanced STING‐VSVG stability compared to untreated controls (Figure 1H). Despite this stabilization, DMXAA stimulation failed to induce TBK1 or IRF3 activation (Figure 1G,H), indicating that STING‐VSVG remained incapable of triggering downstream signaling. Furthermore, mutating the COPII sorting DxE motif within VSVG restored STING trafficking and signaling activity (Figure 1E,G), supporting the proposed functional role of this motif. Intriguingly, immunofluorescence analysis revealed that STING‐VSVG accumulated in lysosomes upon BafA1 treatment (Figure 1D), confirming lysosome‐mediated degradation as the primary mechanism for its turnover. Together, these findings not only highlighted the importance of STING ER export for downstream signal activation (Figure 1D,E), but also emphasized the critical role of the relative strength of COPII anterograde versus COPI retrograde trafficking in controlling STING the early secretary organelle localization (Figure 1A–C).

### 2.2 Structural Characterization of STING pSGME or pFS Motifs Bound to the COPII Coat

The retrieval trafficking of STING has been suggested to involve a COPI‐dependent Surf4 adapter protein,^[^ ^21^ ^]^ however, the signals governing STING's exit from the ER via COPII remain elusive. Since COPII typically recognizes linear sequence motifs, we conducted a sequence alignment of the STING C‐terminal tail (CTT) across 190 mammalian species (Figure 1I). This analysis reveals that the most conserved motifs are concentrated within the C‐terminal segment of the CTT, spanning residues from 354 to 379, which encompass the binding sites for IRF3, TBK1, and clathrin AP1 (Figure 1I,J). The high degree of conservation in this region also suggests the potential presence of COPII sorting motifs. Notably, Figure 1J shows that a canonical COPII sorting ΦC motif—characterized by two hydrophobic residues at the extreme C‐terminus—is present in 164 out of 190 species. In contrast, nearly all primate STING variants lack the ΦC motif but instead terminate in an FS motif in 22 out of 190 species (Figure 1I,J). This raises the question of how COPII sorting is mediated in primate STING. To explore this, we first examined the functional relevance of the STING ΦC motif. Given the generally weak interaction affinity between the COPII coat and its sorting signals, we utilized a previously established crystal soaking strategy to map potential interaction.^[^ ^18^, ^40^ ^]^ Specifically, a 10‐mer peptide containing the mouse STING ΦC motif was soaked with the Sec23a/24a/22b complex crystal, in which the B site is available for binding. Subsequent structural determination revealed the clear *Fourier* difference electron density corresponding to the binding of the mouse STING ΦC motif to the Sec24a B site (Figure S1E–G, Supporting Information), consistent with our previous findings.^[^ ^18^ ^]^ To further assess the functional role of the mouse STING ΦC motif, we introduced two single point mutations (L377W or I378W) that disrupt the ΦC motif without affecting either TBK1 or IRF3 binding (Figure 1I).^[^ ^41^, ^42^, ^43^ ^]^ Functional assays demonstrated that both mutations activated TBK1; however, the L377W mutant completely failed to induce IRF3 phosphorylation, while the I378W mutant significantly attenuated IRF3 phosphorylation upon 50 µM DMXAA treatment (Figure S1C,D, Supporting Information). These findings indicated that the STING ΦC motif is critical for proper STING signal transduction.

To further investigate the potential COPII sorting motifs on human STING, we synthesized a 25‐mer peptide corresponding to the CTT of human STING, spanning residues from 354 to 379 (Figure 1I,J). However, no *Fourier* difference electron density corresponding to the peptide was detected in the map, suggesting the absence of detectable binding between human STING CTT and the COPII coat using this approach. Given that cGAMP binding induces STING trafficking out of the ER via COPII vesicles, we hypothesized that activated human STING, particularly in its phosphorylated forms, may harbor novel COPII‐recognized signals. This hypothesis was informed by two key observations: 1) The B sites of Sec24 are characterized by a cluster of highly positively charged residues, including R430, R435, R750, and R752, which form a critical binding pocket for recognizing the carboxyl group of protein C‐termini in the ΦC or glutamate residue within LxxLE motif interactions.^[^ ^17^, ^18^ ^]^ This structural feature of the Sec24 B site presents an ideal pocket for binding phosphorylated motifs.^[^ ^45^, ^46^ ^]^ 2) It is well established that multiple sites on the STING CTT, including S366, T376, and S379, are phosphorylated by TBK1 following activation.^[^ ^42^, ^47^ ^]^ Notably, the phosphorylation of S366 creates an IRF3‐binding pLxIS motif, which facilitates IRF3 phosphorylation through TBK1, with STING acting as a scaffold to enhance this process.^[^ ^43^, ^48^ ^]^

To explore potential COPII sorting motifs within the phosphorylated CTT of human STING, we synthesized four peptides corresponding to known phosphorylated sites: S366, T376, and S379. Three peptides represented individual phosphorylated residues, while the fourth peptide contained both T376 and S379 as doubly phosphorylated residues (Table S1 (Supporting Information), and Materials and Methods). Using the crystal soaking strategy for structure determination, the *Fourier* difference electron density maps clearly identified two phosphorylated peptides bound to Sec24 B site, including the pSGME and pFS motifs (Figure 1L,N; Table S1, Supporting Information). In the pSGME‐Sec24 complex structure, the phosphate group of phosphorylated STING S366 forms ionic bonds with the guanidinium groups of Sec24 residues R752 and R750. The side chain of STING M368 engages in hydrophobic interactions with Sec24 residue Y436. And the STING E369 interacts with Sec24 residue R750. While STING G367 does not directly participate in the interaction with Sec24, its flexible conformation facilitates the binding of pS366 and M368 to Sec24 (Figure 1L,M). Similarly, in the pFS‐Sec24 complex structure, the phosphate group of phosphorylated S379 is recognized by the guanidinium groups of Sec24 residues R752 and R430, while the side chain of STING F378 docks into a hydrophobic cleft formed by Sec24 residues L498 and V748 (Figure 1N,O). The comparison between the phosphorylated pSGME and pFS motifs of SITNG and the canonical COPII‐binding ΦC and DxE motifs reveals that both phosphorylated motifs closely resemble these established COPII sorting motifs (Figure 1P–S). Notably, the involvement of the carboxylate group in the pFS motif, which binds to Sec24 residue R430 (Figure 1O,R–S), suggests that the COPII sorting signal of the pFS motif requires the final serine residue to be positioned at the extreme C‐terminus of the protein. Although the replacement of the C‐terminal residue with serine typically disrupts the canonical ΦC motif,^[^ ^18^ ^]^ our findings demonstrated that phosphorylated serine can generate a novel motif recognized by COPII, effectively mimicking the ΦC motif. To further confirm the interaction between these phosphorylation motifs and Sec24 coat, we first generated STING mutants including STING FD or DGME, in which the S residue was mutated to phosphorylation mimicking D residue. Co‐IP experiments demonstrated that both of these two STING mutants increased the affinity to Sec24 (Figure S1K, Supporting Information). Secondly, we employed the GST pull‐down experiments to directly examine the association between the GST‐tagged phosphorylated STING cytosolic domain and Sec24a, showing that the phosphorylation on STING evidently increased its Sec24a interaction (Figure S1L, Supporting Information). This discovery highlighted the essential role of phosphorylation at STING S379 residues in generating new COPII sorting signals on activated STING, suggesting that STING phosphorylation might act as a molecular switch regulating its export from the ER.

### 2.3 pSGME and pFS Mutations of STING Impair STING Trafficking and Suppresses Downstream Signaling Activation

**Figure 2:**
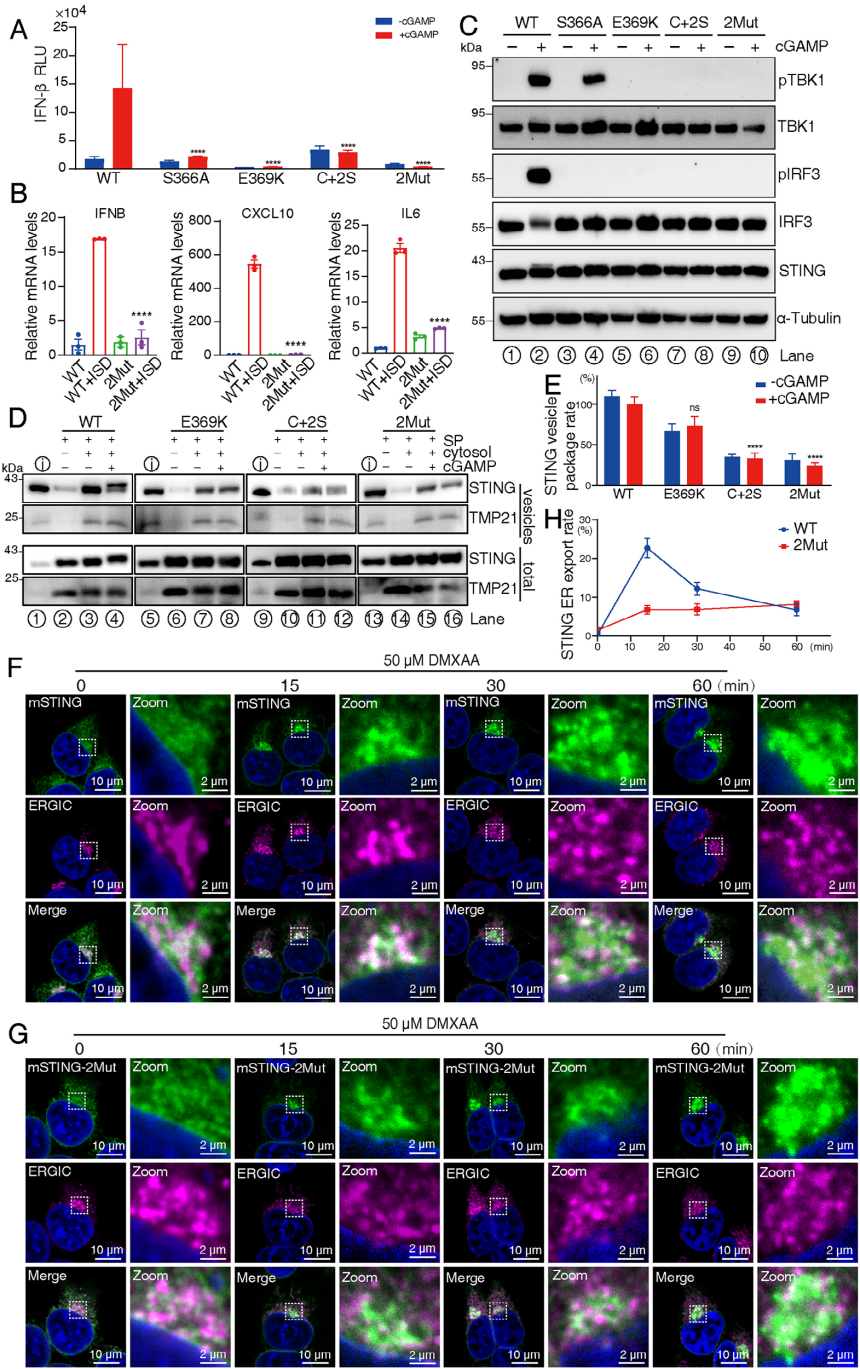
Impaired STING trafficking and activation resulting from structural mutagenesis. A) Analysis of downstream signaling in STING mutants (S366A; E369K; C+2S: two serine residues are added at the STING C‐termini) using the IFN‐β luciferase reporter assay. Data is presented as mean ± SEM after analysis of two‐way ANOVA, *n* = 3. ****P < 0.0001, compared to the WT + cGAMP group. B) The bar graph shows the relative expression of *IFNB*, *CXCL10*, and *IL‐6* in HEK‐293T cells that stably express either WT STING or 2Mut after co‐transfection with ISD and cGAS. Data is presented as mean ± SEM after analysis of one‐way ANOVA, *n* = 3. ****p < 0.0001, compared to the WT + ISD group. C) Western blot analysis of TBK1 activation and IRF3 phosphorylation in STING mutants, with or without treatment with 5 µM cGAMP for 1 h. D, E) Packaging efficiency of STING WT and mutants into COPII vesicles, analyzed via Western blot (D), with quantitative calculation of packaging efficiency (E). Data is presented as mean ± SEM after analysis of two‐way ANOVA, *n* = 3. ****p < 0.0001, ns indicates no significant difference, and data is compared to the WT STING activated by cGAMP treatment group. The COPII vesicle packaging efficiency of STING and its mutant variants was determined by calculating the percentage of STING levels incorporated into vesicles relative to its input STING levels, with normalization to WT packaging ratio. F–H) Representative confocal microscopy images showing the trafficking of WT and 2Mut STING to the ERGIC (F‐G), and the colocalization of STING with ERGIC quantified using 30 HEK‐293T cells (H). ER export efficiency of STING by measuring the ratio of STING fluorescence colocalized with ERGIC to the total cellular STING fluorescence intensity.

Intriguingly, the pFS motif is dominantly conserved in primates, and the pSGME and pSGMD motifs are highly conserved across almost all mammalian species (Figure 1I). To examine whether the pSGMD motif is also a COPII sorting signal, we also solved the complex structure of mouse STING pSGMD bound to Sec23a/24a/22b, and the binding mode of pSGMD motif to COPII is similar to pSGME motif (Figure S1H–J, Supporting Information). For simplicity, we refer to the pSGME/D motif as pSGME here. The conservation of the pSGME motif suggests that the TBK1‐controlled phosphorylation COPII sorting motif may generally be responsible for STING ER export trafficking triggered through cGAMP binding in all species. To further explore this hypothesis, we designed mutations on human STING to disrupt these phosphorylated motifs without interfering with TBK1 or IRF3 binding, based on the complex structures of STING with TBK1 (PDB ID: 6O8C) and IRF3 (PDB ID: 5JEJ) (Figure S2A–C, Supporting Information).^[^ ^42^, ^43^ ^]^ These mutations included E369K and the addition of two serine residues to the STING C‐terminus, referred to as the STING C+2S mutant. The E369K mutation attenuates the pSGME motif, while the C+2S mutation abolishes the ΦC‐like motif of pFS (Figure 1L–O).^[^ ^18^ ^]^ In the IFN‐β luciferase reporter assay, the STING E369K, C+2S, and double mutants all failed to activate the reporter (**Figure** **2**A), similar to the well‐characterized negative control STING S366A mutant, which disrupts the interaction between STING and IRF3.^[^ ^43^ ^]^ Furthermore, gene expression analysis via RT‐qPCR revealed significant downregulation of *IFNB*, *CXCL10*, and *IL6* in HEK‐293T cells, which stably expressed the STING 2Mut compared to WT STING when co‐transfected with ISD and cGAS (Figure 2B). These observations were corroborated by Western blot analysis of downstream signaling pathways, which confirmed that the disruption of these phosphorylated motifs resulted in the inactivation of TBK1 and IRF3 signaling both in human and mouse STING (Figure 2C; Figure S2F–H, Supporting Information).

In whole cells, STING undergoes continuous trafficking through both COPI and COPII vesicles (Figure 1),^[^ ^21^, ^49^, ^50^, ^51^ ^]^ making it challenging to isolate and study the specific process of STING packaging into COPII vesicles. To overcome this limitation, we employed a cell‐free COPII vesicle reconstitution system, which selectively reconstitutes the COPII vesicles without the involvement of COPI vesicles.^[^ ^52^, ^53^ ^]^ This approach enabled us to exclusively investigate the mechanisms underlying STING packaging into COPII vesicles (see Materials and Methods for experimental details). Following reconstitution, vesicles, and cellular organelles were separated through differential centrifugation, and their components were analyzed via Western blot analysis. The abundance of COPII vesicles was calibrated using the marker protein TMP21.^[^ ^18^ ^]^ The STING mutations significantly reduced the packaging efficiency of COPII vesicles compared to WT STING. Specifically, the E369K mutation reduced packaging by ≈35%, while the C+2S and 2Mut attenuated packaging by ≈70% relative to WT STING (Figure 2D,E). Importantly, these STING mutations did not alter the overall abundance of COPII vesicles, indicating that the mutations did not affect COPII vesicle production or function (Figure 2D).

To further confirm the critical roles of these COPII trafficking motifs, we investigated the trafficking of mouse STING following 45 min of treatment with 50 µM DMXAA in a time‐course experiment. Notably, mouse STING retains both the pSGME and canonical ΦC motifs (Figure 1G,H). We calculated the colocalization rates of mouse STING with the ERGIC, the first trafficking station for STING export from the ER. The trafficking rate of mutated mouse STING (mSTING‐2Mut: D368K and I378W) was slower than that of WT STING at the 15‐ and 30‐min time points, suggesting that defective packaging of STING into COPII vesicles delays its ER export (Figure 2F–H). Interestingly, zebrafish STING predominantly activates the NF‐κB pathway, in contrast to the canonical TBK1/IRF3 pathway typically observed in mammalian STING. Sequence analysis revealed that the pSGME and pFS motifs, which are critical for mammalian STING function, are absent in the C‐terminal tail (CTT) of zebrafish STING (Figure S2E, Supporting Information). To investigate the functional significance of these motifs, we generated a chimeric STING construct by replacing the mouse STING CTT with that of zebrafish. Time‐course analysis of STING trafficking following DMXAA treatment revealed that the chimeric STING exhibited a slower trafficking rate compared to WT STING (Figure S2D,E, Supporting Information). Furthermore, we examined the function of mutations in mouse STING, including E368K, which disrupts the pSGME motif, and I378W, which abolishes the ΦC motif. While the E368K mutation and the double mutation (E368K/I378W) completely abrogated IRF3 phosphorylation, the I378W mutation significantly reduced IRF3 phosphorylation, similar to human STING (Figure S2F–H (Supporting Information) and Figure 2C). Our findings were consistent with our structural studies (Figure 1H–L), further validating the critical role of these motifs in STING trafficking and activation.

### 2.4 Phosphorylated STING Sorting into COPII Vesicles Drives Compartmentalized Activation of Downstream Signaling

**Figure 3:**
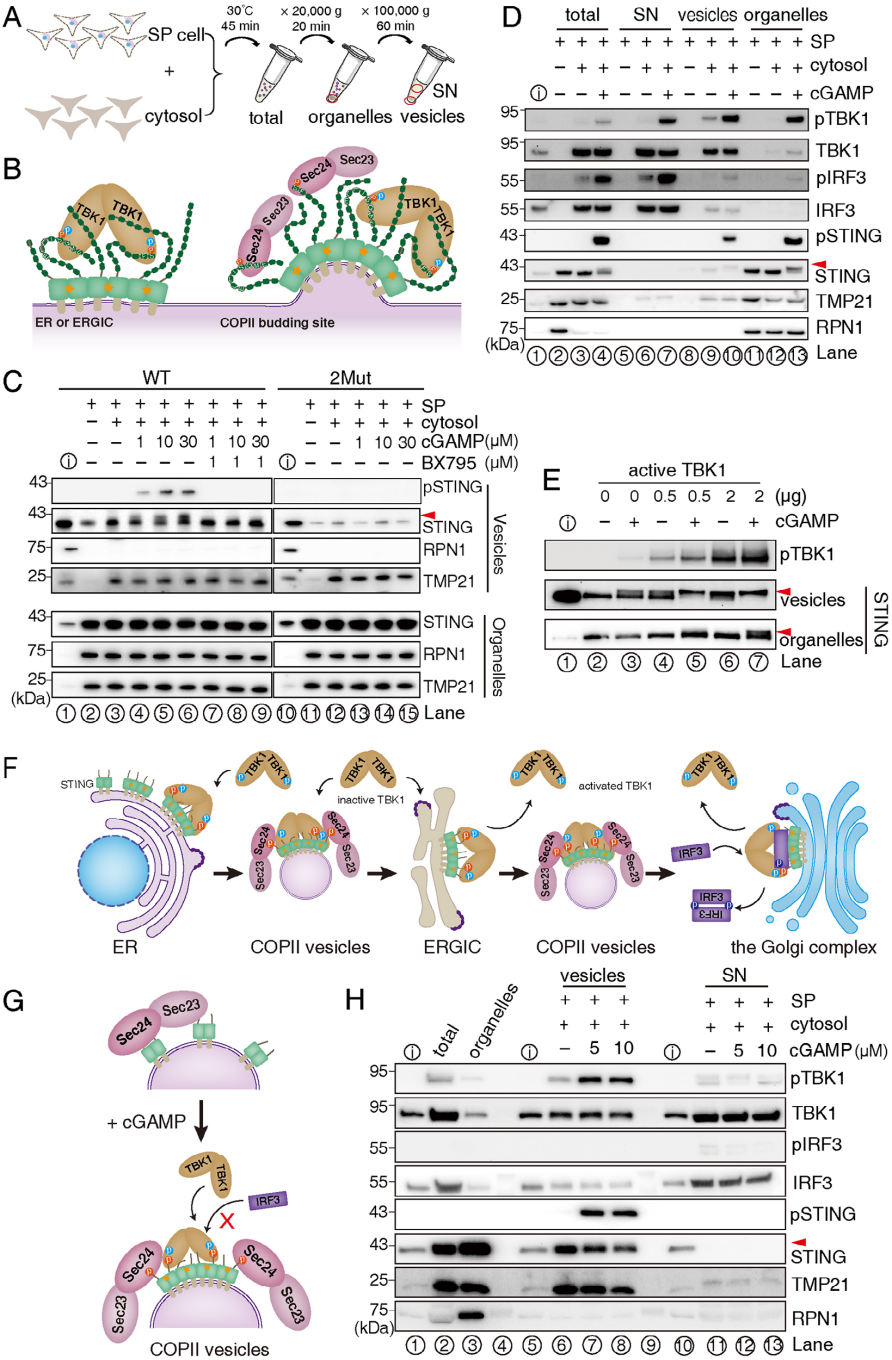
Selectively packaging of phosphorylated STING into COPII vesicles and spatial activation of TBK1 and IRF3 signaling using a cell‐free COPII vesicle reconstitution approach. A) Schematic representation of the cell‐free reconstitution strategy for COPII vesicle formation. B) Diagram illustrating the recognition of pSGME and pFS motifs by Sec24, leading to the packaging of both phosphorylated and unphosphorylated STING into COPII vesicles. C) Western blot analysis of STING packaging into COPII vesicles. ⓘ Denotes 1% input. Phosphorylated STING is indicated by a red triangle. Sec22 serves as a COPII vesicle marker, while RPN1 is used as an ER marker. D) Western blot analysis showing the fractional distribution of active TBK1, phosphorylated IRF3, and STING in the supernatant (SN), COPII vesicles, and cellular organelles. E) Active TBK1 phosphorylates STING on the ER, and the phosphorylated STING is selectively packaged into COPII vesicles. F) Schematic diagram of the spatial distribution of STING signaling. Following cGAMP‐mediated activation of STING, inactive TBK1 is recruited to either COPII vesicles or the ERGIC, where it becomes activated. A portion of the activated TBK1 is released from COPII vesicles or ERGIC into the cytosol, where it further phosphorylates STING on organelles. The pSGME and pFS motifs facilitate STING packaging into COPII vesicles. IRF3 is not recruited to vesicles due to Sec24's interaction with overlapping binding sites. IRF3 activation occurs exclusively at the ERGIC or *cis*‐Golgi complex following COPII vesicle uncoating. G, H) STING localized on COPII vesicles activates TBK1 but fails to recruit or phosphorylate IRF3.

To further elucidate the trafficking mechanisms and downstream signaling activation of STING, we employed the cell‐free COPII vesicle reconstitution system to first assess the packaging efficiency of phosphorylated STING (**Figure** **3**A). Notably, the phosphorylated STING exhibited a preferential incorporation into COPII vesicles compared to its unphosphorylated counterpart, which remained the predominant form within cellular organelles under cGAMP treatment across a concentration gradient (Figure 3B,C, compare STING bands lane 3–6 between vesicles and organelles fraction). This phosphorylation event was mediated by TBK1 activation, as evidenced by the complete abrogation of STING phosphorylation upon treatment with a specific TBK1 inhibitor, BX795 (Figure 3C, lane 7–9). Meanwhile, the lower packaging efficiency of STING‐2Mut compared to WT STING further supports the role of phosphorylation in regulating COPII sorting (Figure 3C, lanes 10–15). These findings aligned with our structural and mutagenesis studies, which revealed that the pSGME and pFS motifs generated novel COPII sorting signals, thereby enhancing the efficiency of ER export relative to the native, unphosphorylated form of STING.

As shown in Figure 1B,F, TBK1 remained inactive when STING was retained at the ER due to the presence of a robust ER retention signal, WDM, even in the presence of cGAMP. This observation is consistent with a previous report,^[^ ^54^ ^]^ suggesting that STING localized to the ER is incapable of activating TBK1. Given that COPII coats facilitate vesicle budding from both the ER and the ERGIC, it is essential to delineate where phosphorylated STING is sorted into COPII vesicles and where subsequent downstream signaling events are initiated. To address this, upon completion of the COPII vesicle reconstitution reaction, the mixture was fractionated into three distinct components: purified reconstituted COPII vesicles, semi‐permeable (SP) cell membranes (primarily composed of cellular organelles), and the supernatant (SN: representing cytosolic material) (Figure 3A). First, we observed that STING present in the SP cell membranes underwent oligomerization upon cGAMP treatment, as demonstrated by native gel electrophoresis (Figure S3A,B). This finding indicated that STING within the SP membranes remains functionally active. Further, treatment with cGAMP during the reaction effectively induced TBK1 activation, and IRF3 phosphorylation, thereby validating the physiological relevance of our experimental approach (Figure 3D, lane 1–4). Subsequent molecular distribution analysis revealed that active TBK1 was detected in multiple fractions, including COPII vesicles, SP cell membranes, and SN (Figure 3D, lane 7, 10, 13). This observation indicated that a portion of active TBK1 dissociated from the membrane‐bound STING and was released into the cytosol, suggesting a dynamic redistribution of TBK1 following its activation. In contrast, phosphorylated IRF3 was predominantly localized in the cytosolic fraction, suggesting that upon phosphorylation, IRF3 dissociated from the STING‐TBK1 complex (Figure 3D, lane 7). This observation was consistent with its established physiological role, wherein phosphorylated IRF3 translocated to the nucleus to initiate transcriptional activation.^[^ ^48^ ^]^

Because of the presence of active TBK1 in the cytosolic fraction, we sought to determine whether cytosolic TBK1 could phosphorylate STING localized on the ER. To this end, SP cell membranes, prepared from HEK‐293T cell stably expressing WT human STING, were incubated with a concentration gradient of recombinant active TBK1 (Figure S3C), which was expressed and purified from insect cells.^[^ ^55^ ^]^ Our results demonstrated that recombinant active TBK1 efficiently phosphorylated ER‐localized STING (Figure 3E, lane 2, 4, 6), leading to its enhanced packaging into COPII vesicles compared to WT STING (Figure 3E, lane 2, 4, 6). These findings established that cytosolic active TBK1 phosphorylated STING on the ER (Figure 3F), suggesting that phosphorylated STING sorted into COPII vesicles could happen in the ER.

To determine whether STING localized on COPII vesicles retained the ability to initiate downstream signaling, we purified COPII vesicles containing STING in the absence of cGAMP and subsequently incubated these vesicles with 5 or 10 µM cGAMP for 15 min. Intriguingly, TBK1 was robustly activated by STING on COPII vesicles, whereas no activation was observed in the cytosolic fraction, demonstrating that TBK1 activation specifically occurs on STING COPII vesicles (Figure 3H, lane 2, 5–8, 10–13). In contrast, IRF3 phosphorylation was not detected in this system (Figure 3H). This observation can be plausibly explained by the competitive interaction of Sec24 with the phosphorylated pSGME motifs, which share the pS366 site with the IRF3‐binding pLxIS motif, potentially impeding the recruitment of IRF3 to COPII vesicles (Figure 1I,J and Figure 3G). To validate this, GST pull‐down experiments were conducted to assess the competitive binding between Sec24 and IRF3 to the CTT of STING. As shown in Figure S3D, Sec24 significantly attenuated the interaction of IRF3 with the phosphorylated STING CTT, suggesting that IRF3 recruitment to COPII vesicles is hindered while COPII coats remain assembled. Collectively, these results suggested that while TBK1 activation took place on COPII vesicles, IRF3 phosphorylation likely occurred at later stages, such as the ERGIC or the *cis*‐Golgi complex, where COPII vesicles underwent coat dissociation and fusion with these organelles (Figure 3F,G). This spatial segregation of signaling events underscored the compartmentalized nature of STING‐mediated immune activation.

### 2.5 A Class of 4‐PBA‐Like Natural Compounds Tune Down IRF3 Activation Through Attenuating STING COPII Trafficking

Our previous biochemical and structural studies identified the molecular chaperone 4‐phenylbutyric acid (4‐PBA) as an interactor with the B site of the Sec24 coat, resembling the binding mode of the ΦC motif.^[^ ^18^ ^]^ 4‐PBA is an FDA‐approved drug currently used as adjunctive therapy for chronic urea cycle disorders in children.^[^ ^56^ ^]^ Over‐activation of the STING signaling pathway will lead to dozens of human diseases, including, AGS,^[^ ^5^ ^]^ SAVI,^[^ ^6^ ^]^ and COPA syndrome.^[^ ^49^ ^]^ Given this, we sought to investigate whether inhibiting STING trafficking out of the ER could reduce its downstream overactivation, thereby identifying a potential therapeutic strategy for these diseases.

**Figure 4:**
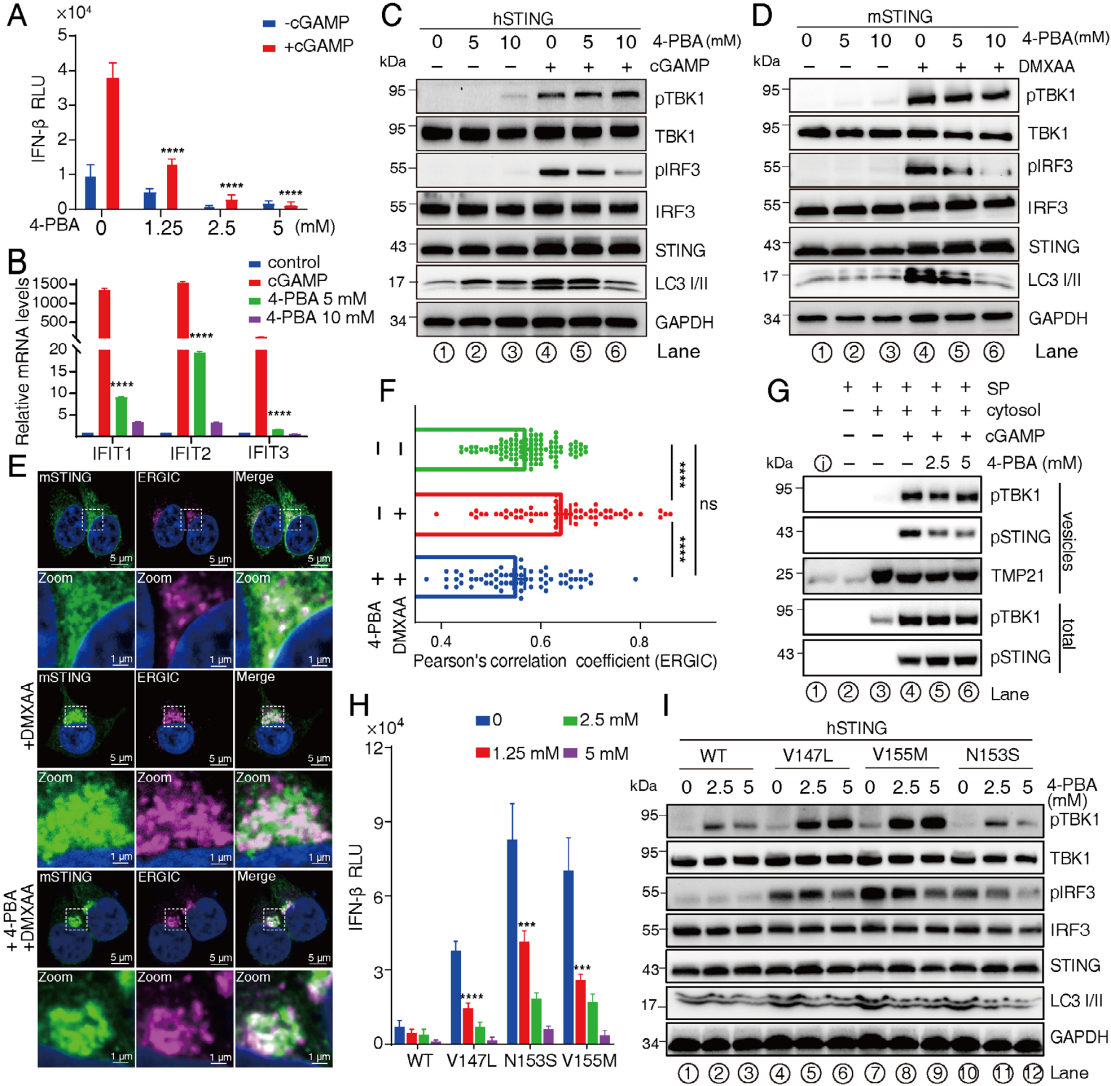
4‐PBA attenuates STING‐mediated IRF3 activation by competing with STING for Sec24 B sites. A) 4‐PBA inhibits the IFN‐β luciferase reporter response in a concentration‐dependent manner. Data is presented as mean ± SEM after analysis of two‐way ANOVA, *n* = 3. ****P < 0.0001, compared to the 0 mM 4‐PBA + cGAMP group. B) 4‐PBA reduces the expression of *IFIT1‐3*, as measured by RT‐qPCR. Data is presented as mean ± SEM after analysis of one‐way ANOVA, *n* = 4. ****P < 0.0001, compared to the cGAMP group. C,D) 4‐PBA treatment does not affect TBK1 activation but significantly inhibits IRF3 phosphorylation. E,F) 4‐PBA delays STING export from the ER, with rates calculated based on the colocalization of STING with ERGIC. Data is presented as mean ± SEM after analysis of one‐way ANOVA, *n* = 60. ****P < 0.0001, ns indicates no significant difference. G) Adding 4‐PBA reduced the packaging of phosphorylated STING in COPII vesicles. H,I) 4‐PBA attenuates hyperactivation of SAVI‐associated STING mutants, as demonstrated by IFN‐β luciferase reporter assays (G) and Western blot analysis (H). Data is presented as mean ± SEM after one‐way ANOVA analysis, *n* = 3. ****P < 0.0001, compared to 0 mM 4‐PBA.

To this end, we evaluated the ability of 4‐PBA to attenuate STING activation. Treatment with a concentration gradient of 4‐PBA significantly reduced the activation of IFN‐β, as demonstrated by luciferase reporter assays (**Figure** **4**A; Figure S4A, Supporting Information). Additionally, we monitored the expression of IRF3‐regulated genes using RT‐qPCR and found that 4‐PBA treatment effectively reduced the expression levels of *IFIT1‐3* (Figure 4B). To further validate these findings, we analyzed TBK1 and IRF3 signaling in HEK‐293T cells stably expressing either human or mouse STING. While TBK1 activation remained intact (Figure 4C, lane 4–6 and 4D, lane 4–6), the phosphorylation of IRF3 was markedly attenuated and the autophagosomal marker LC3‐II expression was also inhibited upon 4‐PBA treatment (Figure 4C, lane 4–6 and 4D, lane 4–6), consistent with the results from the luciferase reporter assays (Figure 4A). The autophagy pathway is linked to COPII‐dependent STING trafficking, a process intrinsically connected to autophagosome membrane biogenesis and antimicrobial defense mechanisms.^[^ ^57^, ^58^, ^59^, ^60^, ^61^, ^62^ ^]^ Notably, this regulatory pathway was modulated by 4‐PBA treatment (Figure 4C, lane 4–6; Figure 4D, lane 4–6). Further, in HeLa and K562 cells, both of which endogenously express STING, we also discovered that the treatment of 4‐BPA reduced the STING downstream signals (Figure S4C–E, Supporting Information). Additionally, upon the 45 min treatment of 50 µM DMXAA with HEK‐293T cell with stable expression of mouse STING, we quantitatively measured the DMXAA‐activated STING trafficking rate reduction by 4‐PBA treatment and revealed that 4‐PBA significantly turned the DMXAA‐activated STING trafficking to ERGIC than control (Figure 4E,F). This attenuation of STING ER export trafficking by the treatment of 4‐PBA was further confirmed by the COPII vesicle reconstitution assays, which showed that the addition of 4‐PBA in the reaction significantly reduced the packaging efficiency of phosphorylated STING, confirming the whole‐cell results (Figure 4G). Additionally, to investigate whether 4‐PBA influences the ability of STING to bind to its agonist, we treated HEK‐293T cells stably expressing mouse STING with DMXAA. The polymerization profile of mouse STING showed no significant changes compared to the control, indicating that 4‐PBA did not impair DMXAA binding to STING (Figure S4B, Supporting Information). Furthermore, treatment with 4‐PBA did not alter the degradation profile of DMXAA‐activated endogenous STING in RAW264.7 cells over a time course of 1 to 3 h, suggesting that while 4‐PBA regulates STING ER export trafficking, it does not affect the final degradation fate of STING (Figure S4F, Supporting Information).

The SAVI syndrome is a rare autoinflammatory disorder driven by gain‐of‐function mutations in STING, such as V147L, N153S, and V155M.^[^ ^6^ ^]^ Currently, the only promising therapeutic option in clinics is the Janus kinase (JAK) inhibitors.^[^ ^63^, ^64^ ^]^ We evaluated the potential of 4‐PBA to counteract the hyperactive signaling associated with SAVI‐linked STING mutants, focusing on the V147L, N153S, and V155M variants. The V147L and V155M mutants displayed significantly elevated activity compared to WT STING, while N153S showed similar TBK1 activity and marginally increased IRF3 phosphorylation (Figure 4H,I),^[^ ^65^ ^]^ and treatment with 4‐PBA significantly suppressed this hyperactivity (Figure 4H,I). Further investigation of STING downstream signals demonstrated that TBK1 activity remained unaltered (Figure 4I, lanes 4–12), whereas IRF3 phosphorylation was progressively inhibited in a dose‐dependent manner with increasing 4‐PBA concentrations (Figure 4I, lane 4–12). Moreover, the treatment of 4‐PBA clearly attenuated the trafficking rate of V155M STING slower than the control, suggesting the downregulation of IRF3 phosphorylation caused by 4‐PBA‐mediated STING trafficking defection (Figure S4G,H, Supporting Information). These results suggest that 4‐PBA attenuates STING‐mediated signaling by specifically targeting IRF3 activation, likely through its ability to modulate STING trafficking. This underscored the therapeutic potential of 4‐PBA in addressing STING‐driven disease.

Furthermore, we observed that a class of natural compounds derived from phenylpropanoids, specifically hydrocinnamic acid derivatives, exhibit structural similarities to 4‐PBA. These compounds are known to comprise a series of bioactive components with significant anti‐inflammatory, antioxidant, and immunomodulatory properties, particularly as documented in traditional Chinese medicine.^[^ ^66^, ^67^, ^68^ ^]^ Given these characteristics, we investigated whether this class of natural compounds could modulate the cGAS‐STING signaling pathway.

To evaluate this, we employed the luciferase IFN‐β reporter assay to screen ten distinct hydrocinnamic acid derivatives and three short‐chain fatty acids (SCFAs) which originate from human gut microbiota metabolism also with a structural similar feature as 4‐PBA (Figure S5, Supporting Information).^[^ ^69^ ^]^ The results revealed that ferulic acid, caffeic acid, 4‐bromocinnamic acid, 4‐phenylcinnamic acid, and trans‐2,5‐difluorocinnamic acid all significantly attenuated IFN‐β reporter activity (Figure S5A–M, Supporting Information). Notably, ferulic acid and caffeic acid, both of which have been previously reported to exhibit anti‐inflammatory properties,^[^ ^68^, ^70^ ^]^ demonstrated potential involvement in the inhibition of the STING pathway. To further validate these findings, we performed a Western blot analysis to assess the phosphorylation status of IRF3. The results demonstrated a concentration‐dependent reduction in IRF3 phosphorylation levels in response to ferulic acid treatment (Figure S5N, lane 4–6 (Supporting Information)). Additionally, quantitative analysis of STING‐mediated signaling revealed the downregulation of downstream effector genes, including *IFIT1‐3, IFNB*, *IL6*, *NF‐κB*, *CXCL10* and *TNF‐a* further supporting the inhibitory effects of ferulic acid on the STING downstream pathway (Figure S5O–V, Supporting Information). Our findings indicated that these 4‐PBA‐like natural hydrocinnamic acid derivatives are capable of downregulating the STING pathway, thereby offering a promising strategy for the discovery of novel STING signaling inhibitors in future research.

## 3 Discussion

### 3.1 STING pSGME and pFS: the First Identified Phosphorylated COPII Sorting Motifs

Phosphorylated motifs are well‐documented in the desensitization of cell surface receptors, serving as key signals for recruiting adaptor proteins like β‐arrestins to facilitate clathrin‐mediated endocytosis.^[^ ^71^, ^72^ ^]^ This process removes active receptors from the cell surface and directs them to endosomal compartments for degradation, recycling, or signal modulation. By utilizing phosphorylated motifs, cells achieve precise control over receptor responsiveness, preventing excessive or prolonged activation.^[^ ^71^, ^72^ ^]^ In this study, we uncovered a novel role for phosphorylated motifs in STING signal regulation, identifying the first COPII‐recognized phosphorylated motifs generated upon cGAMP activation (Figure 1F–L). In contrast to the phosphorylated motifs of surface receptors, the pSGME and pFS motifs of human STING are responsible for the proper initiation of downstream signaling. Notably, the identification of the pSGME COPII sorting motif aligns with STING post‐Golgi trafficking mediated by clathrin AP1, as both AP1 and COPII share the same key binding residue, pS366.^[^ ^24^ ^]^ This observation provided a coherent explanation for the entire trafficking process of STING. Interestingly, although the primary functions of phosphorylated motifs differ between surface receptors and STING on the ER, their ultimate destinations—endosomes or lysosomes for degradation—are remarkably similar.

While the pFS motif of STING is conserved only in primates, the pSGME motif is highly conserved across all species. This suggests that our findings regarding the sequential and spatial activation of STING downstream signaling through phosphorylation‐regulated COPII trafficking are likely conserved across species. Furthermore, structural analysis of the pSGME and pFS motifs revealed that these phosphorylated motifs resemble the ΦC motif, which binds to the B site of Sec24. Notably, the presence of a canonical ΦC motif in non‐primate STING and the absence of the ΦC motif in primate STING indicates a tighter finely tuned mechanism for primate STING trafficking and activation than non‐primtates’.^[^ ^73^, ^74^ ^]^ Furthermore, evolutionary analysis reveals that the canonical COPII sorting ΦC motif in non‐primates has been replaced by the pFS motif in primates. This substitution suggests two important functional implications: first, STING containing the ΦC motif may exhibit slightly enhanced basal COPII‐mediated ER export compared to the primate pFS variant in the inactive state; second, the phosphorylation‐switchable nature of the pFS motif indicates that the coupling between STING activation and trafficking may be subject to more stringent regulation in primates compared to other species.

### 3.2 Sequential Activation Hubs for TBK1 and IRF3 are Built by Phosphorylation‐Regulated STING COPII Trafficking

Currently, it is established that the binding of cGAMP to STING results in the dissociation between STING and its COPI retrieval trafficking adapter, Surf4.^[^ ^21^ ^]^ Therefore, cGAMP‐bound STING will be released from the ER retention mechanism via COPI. However, whether STING COPII vesicle trafficking is also involved is a long‐standing unknown question in cGAS‐STING field. In this study, we characterized pSGME and pFS motifs as bona fide COPII sorting signals. Notably, STING mutations impairing the pSGME and pFS motifs markedly reduced the packaging efficiency of STING into COPII vesicles, leaving only residual packaging likely driven by bulk flow trafficking (Figure 2C,D).^[^ ^75^ ^]^ This finding highlighted the critical role of these phosphorylated motifs in regulating STING trafficking. Importantly, we showed that TBK1 activation occurs on COPII vesicles, while IRF3 phosphorylation is confined to the ERGIC or *cis*‐Golgi complex after vesicle decoating, due to competitive binding between the COPII coat and IRF3 (Figure 3, Figure S3D). Whole‐cell experiments demonstrated that both these STING mutations and 4‐PBA treatment partially inhibited the anterograde trafficking rate of STING, corroborating these results (Figure 2E–G).

Moreover, we proposed a working model for the trafficking of phosphorylated STING via COPII vesicles. Upon cGAMP‐induced oligomerization, STING activates TBK1, which in turn phosphorylates neighboring STING molecules. This phosphorylation generates novel binding sites for COPII coat sorting on the ER or ERGIC. Through these sites, COPII vesicles transport active TBK1 and STING forward. Once these STING‐containing COPII vesicles fuse with the ERGIC or *cis*‐Golgi complex, the COPII coats detach, allowing IRF3 to be sequentially recruited as a substrate for the active TBK1. In this model, active TBK1 and STING are more selectively packaged into COPII vesicles for trafficking compared to mutants lacking the COPII‐sorting phosphorylated motifs. This selective packaging ensures robust phosphorylation of IRF3 (Figure 3).

Our findings demonstrated that STING underwent dynamic COPI‐ and COPII‐mediated trafficking, which facilitated its residence at the ER in an inactive state and its export from the ER in an active state through TBK1‐controlled phosphorylation of the pSGME and pFS motifs. Our study also showed that STING ER export trafficking assisted in the formation of distinct activation hubs for TBK1 and IRF3 along the early secretory pathway.

### 3.3 Identification of Natural Compounds Down‐Regulating the STING Pathway by Targeting its ER Export

In this study, we demonstrated that 4‐PBA effectively downregulated STING signaling by disrupting proper STING trafficking (Figure 4A–G). Furthermore, our discovery showed significant therapeutic potential of 4‐PBA for SAVI syndrome (Figure 4H–I), suggesting that this FDA‐approved drug could be repurposed to treat this disease. Given the central role of STING in various autoimmune and inflammatory diseases, our findings also raise the possibility of extending the application of 4‐PBA to other STING‐related autoimmune disorders, offering a promising avenue for future clinical interventions.^[^ ^76^ ^]^

Intriguingly, based on the structural similarity between phenolic acids in phenylpropanoids and 4‐PBA, we successfully identified five compounds capable of attenuating the IFN‐β response by regulating STING trafficking (Figure S5A–E, Supporting Information). For example, caffeic acid (CA), a phenolic compound found in various plants, fruits, and beverages such as coffee, has been extensively studied for its anti‐inflammatory and antioxidant properties, particularly its ability to modulate the interferon pathway.^[^ ^77^, ^78^, ^79^, ^80^ ^]^ Additionally, ferulic acid, one of the most abundant phenolic acids in our daily diet and widely used in food supplements and the cosmetic industry, has also been identified. Our study revealed that this class of natural compounds inhibited the STING‐mediated interferon response, providing a mechanistic explanation for the biological function of these important dietary constituents.

Mechanistically, TBK1 activation is closely linked to cGAMP‐induced STING oligomerization.^[^ ^41^ ^]^ In this study, we found that 4‐PBA did not inhibit TBK1 activation following cGAMP treatment (Figure 4C,D), consistent with the observation that 4‐PBA had no effect on cGAMP‐induced STING oligomerization (Figure S4B, Supporting Information). Furthermore, we demonstrated that 4‐PBA modulates the packaging preference of STING into COPII vesicles, thereby influencing IRF3 phosphorylation, a process tightly associated with STING trafficking out of the ER (Figure 4C–G). Specifically, our proposed model suggests that 4‐PBA disrupts the selective incorporation of phosphorylated STING, bound by active TBK1, into COPII vesicles. This disruption reduces the abundance of the active phosphorylated STING and active TBK1 complex at the ERGIC or the *cis*‐Golgi complex, ultimately leading to decreased IRF3 phosphorylation efficiency (Figure 3). Also, our previous work identified Sec24 as the cellular target of 4‐PBA, explaining its known function as an ER stress reducer.^[^ ^18^ ^]^ In this study, we further demonstrate that 4‐PBA can effectively suppress STING overactivation in SAVI mutants, highlighting its therapeutic potential for STING‐associated autoinflammatory disorders. Notably, Sec24a remains the only known cellular target of 4‐PBA, suggesting a high degree of specificity for this compound. While these findings are encouraging, future studies should focus on developing more potent and selective Sec24‐targeting compounds to optimize therapeutic efficacy.

Together, our work not only addressed a long‐standing question regarding the relationship between STING trafficking and its activation but also revealed that compounds derived from our daily diet can effectively mitigate inflammatory responses mediated by STING.

## 4 Experimental Section

### Antibodies and Chemicals

All the antibodies used in this study were purchased as follows: STING Rabbit mAb (CST, Cat: 13647), Phospho‐STING (Ser366) (E9A9K) rabbit mAb (CST, Cat: 50907), TBK1/NAK rabbit polyclonal antibody (CST, Cat:3013), Phospho‐TBK1/NAK (Ser172) (D52C2) rabbit mAb (CST, Cat: 5483), IRF3 rabbit polyclonal antibody (Proteintech, Cat: 11312‐1‐AP), Phospho‐IRF‐3 (Ser396) (4D4G) rabbit mAb(CST, Cat: 4947), TMP21 rabbit polyclonal antibody (Proteintech, Cat: 15199‐1‐AP), Sec22Brabbit mAb (CST, Cat:ab181076), Ribophorin I rabbit mAb (CST, Cat:ab197888), rabbit polyclonal anti‐GM130 (Proteintech, Cat: 11308‐1‐AP), rabbit polyclonal anti‐Sec61β (Proteintech, Cat: 15087‐1‐AP), rabbit polyclonal anti‐ERGIC53 (Proteintech, Cat: 13364‐1‐AP), mouse monoclonal anti‐GAPDH (Proteintech, Cat: 60004‐1‐Ig), goat anti‐rabbit IgG (H+L) secondary antibody, HRP (Invitrogen, Cat: 31460), goat anti‐mouse IgG (H+L) secondary antibody, HRP (Invitrogen, Cat: 31430), goat anti‐rabbit IgG H&L (Alexa Fluor 594) antibody (Abcam, Cat: ab150080).

All the chemicals in this study were purchase as follows: 2′3'‐cGAMP (InvivoGen, Cat: tlrl‐nacga23), DMXAA (InvivoGen, Cat: tlrl‐dmx), Hoechst 33342 (Invitrogen, Cat: H1399), puromycin (InvivoGen, Cat: ant‐pr‐1), Polyethylenimine, Linear, MW 25000, transfection Grade (PEI 25K) (Polysciences, Cat: 23966‐1), polybrene (MERCK, Cat: MKCK6614), Digitonin (MERCK, Cat:D141‐100MG), Creatine Kinase (Roche, Cat: 10127566001), Creatine Phosphokinase (Roche, Cat: 10736988001), Soybean trypsin inhibitor (Chem Cruz, Cat: K1318), Adenosine Triphosphate (Roche,Cat: 10519987001), Guanosine Triphosphate (Roche, Cat: 10106399001), 4‐Phenylbutyric acid (4‐PBA) (MERCK, Cat:P21005), Salvianic acid A (MERCK, Cat: SML0679), Sodium butyrate (MERCK, Cat: 576430), Sodium Valerate (Yuanye, Cat: 6106‐41‐8), Sodium 2‐methylbutanoate (Yuanye, Cat: S35155), Cinnamic acid (Yuanye, Cat: B21082), Caffeic acid (Yuanye, Cat: B20660), Ferulic acid (Yuanye, Cat: B20007), 4‐Bromocinnamic acid (Yuanye, Cat:S41361), 4‐Methoxycinnamic acid (Yuanye, Cat:B25652), 4‐Methylcinnamic acid (Yuanye, Cat:Y77390), trans‐2,5‐Difluorocinnamic acid (Yuanye, Cat: S41088), 4‐Phenylcinnamic Acid (Yuanye, Cat:Y24274), 1,4‐Benzenediacrylic Acid (MERCK, Cat:P23903).

### Plasmid Construction

All expression vectors for insect cells, including the pFastBac1 vector encoding the full‐length humanSec23a, the pFast‐HTB vector encoding the full‐length human Sec24a (lacking residues 1–340), and the pFast‐GST vector encoding the full‐length human TBK1, were derived from our previous study.^[^ ^18^ ^]^ Transformed with pFastBac vectors in DH10Bac cells and generate recombinant bacmids by using the Bac‐to‐Bac baculovirus expression system (Invitrogen).

The C‐terminal tail of human STING (residue number: 335–379) and human STING (residue number: 140–379) were cloned into the pGEX‐6p1 vector (Cytiva, USA), which expresses a protein with N‐terminal fused GST tag for GST pull‐down experiments.

All mammalian expression plasmids were constructed using pLVX‐puro or pcDNA4/To vectors (Invitrogen), expressing target proteins with or without an N‐terminal GFP tag. It was generated: 1) full‐length human and mouse WT STING; 2) mouse trafficking mutants (STING‐WDM [AKEKSD], STING‐WDM‐Mut [AAEASD], STING‐VSVG [QIYTDIEMNRLGK], and STING‐VSVG‐Mut [QIYTAIAMNRLGK]); 3) human phosphomimetic STING (S366D/S379D) along with functional mutants (E369K, C+2S, E369K/C+2S) and SAVI‐associated mutants (V147L, N153S, V155M); 4) mouse COPII sorting mutants (L377W, D368K, I378W, D368K/I378W); and 5) a mouse‐zebrafish chimeric STING where mouse CTT (residues 355–378) was replaced by zebrafish CTT (residues 361–398); as well as 6) full‐length human Sec24A with C‐terminal FLAG tag. All constructs were verified by Sanger sequencing (GENEWIZ, China).

### Cell Culture and Treatment

All mammalian cell lines, including HEK‐293T, HEK‐293FT, HeLa, RAW264.7, and K562, were obtained from the Cell Resource Center at Peking Union Medical College (CRC/PUMC). These cells were maintained at 37 °C in a humidified atmosphere containing 5% CO_2_. HEK‐293T, HEK‐293FT, HeLa, and RAW264.7 cells were cultured in Dulbecco's Modified Eagle's Medium (DMEM, Corning) supplemented with 10% fetal bovine serum (FBS) (Corning, Cat: 35‐015‐CV) and 1% penicillin/streptomycin (Thermofisher, Cat: 10378016). In contrast, K562 cells were cultured in Roswell Park Memorial Institute 1640 medium (RPMI 1640) (Gibco, Cat: 12633020), supplemented with 10% FBS and 1% penicillin/streptomycin. The Sf9 insect cell (Thermofisher, Cat: B82501) and Hi5 (Thermofisher, Cat: B85502) were cultured at 28 °C. Sf9 cells were maintained in SIM SF expression medium (Sino Biological, Cat: MSF1), supplemented with 10% FBS and 1% penicillin/streptomycin, while Hi5 cells were cultured in SIM HF expression medium (Sino Biological, Cat: MHF1), supplemented with 1% penicillin/streptomycin. *Escherichia coli* strains, including DH5α (Thermofisher, Cat: 18265017), BL21(DE3) (Thermofisher, Cat: EC0114), Stbl3 (NEB, Cat: C3040H), and DH10Bac (Thermofisher, Cat: 10361012), were grown in LB medium at 37 °C. The LB medium was supplemented with appropriate antibiotics for plasmid selection at the following concentration: kanamycin (50 µg mL^−1^), ampicillin (100 µg mL^−1^), gentamicin (7 µg mL^−1^), and tetracycline (10 µg mL^−1^).

The method for delivering cGAMP into cells was adapted from a previously published protocol.^[^ ^58^ ^]^ Briefly, cells were first permeabilized using 10 µg mL^−1^ digitonin in Buffer A (50 mM HEPES‐KOH pH 7.2, 100 mM KCl, 3 mM MgCl_2_, 0.1 mM DTT, 85 mM Sucrose, 0.2% BSA, 1 mM ATP) and then the cells were treated by cGAMP (5 µM) for 1 h. Following this treatment, cells were processed for activation analysis by Western blot. In 4‐PBA inhibition experiments, cells were pretreated with specified concentrations of 4‐PBA for 2 h prior to stimulation with either cGAMP or DMXAA.

### Recombinant Proteins Production

In accordance with previously described methods,^[^ ^18^ ^]^ the COPII coat proteins Sec23a/Sec24a (lacking residues 1–340) were purified using an insect cell expression system. Briefly, Hi5 cells were infected with baculovirus, which was produced in Sf9 cells. After infection, the cells were harvested and resuspended in a binding buffer (50 mM Tris‐HCl, pH 8.0, 0.5 M NaCl). The Sec23a/Sec24a proteins were sequentially purified using HisTrap column (Cytiva, Cat: 17 524 802), ion‐exchange chromatography (Cytiva, Cat: 17 524 802), and size‐exclusion chromatography (Cytiva, Cat: 28 989 335). Sec22 (lacking residues 1–195) was expressed in BL21 cells and then purified through a similar protocol. All purified COPII proteins were stored in a buffer containing Tris‐HCl (20 mM, pH 7.4), NaCl (0.2 M), and DTT (2 mM). TBK1 with an N‐terminal GST tag was expressed and purified in the insect cell system. Briefly, Hi5 cells were first infected by the baculovirus, and then the cells were harvested, resuspended in PBS buffer, and then lysed by sonication. The lysate was centrifuged at 50000 × *g* for 2 h at 4 °C, and the supernatant was subject to glutathione Sepharose beads (Cytiva, Cat: 17075605). The GST‐tagged TBK1 protein was eluted using 20 mM reduced glutathione. The eluted protein was then concentrated and desalted into a storage buffer containing Tris‐HCl (20 mM, pH 7.4), NaCl (0.2 M), and DTT (2 mM). All the proteins were flash‐frozen in liquid nitrogen and then stored at −80 °C for future use.

### Cell Transfection and Stable Cell Lines Generation

Cell transfection was conducted using 1 mg mL^−1^ PEI following the manufacturer's instructions. Briefly, the ratio for DNA to PEI is set as 3:1, and the DNA and PEI were first separately diluted in Opti‐MEM medium (Gibco), and then mixed the DNA and PEI solution together, and incubated at room temperature for 15–20 min to allow the formation of DNA‐PEI complexes. The mixture was then carefully dropwisely added to the cells. After 6 h post the transfection, the transfection medium was replaced with a fresh culture medium. Cells were harvested for subsequent experiments 24–48 h post‐transfection.

Stable cell lines were generated by the lentiviral infection strategy. The lentivirus was produced by co‐transfecting HEK‐293FT cells with the pLVX‐Puro vectors and lentiviral packaging vectors (psPAX2 and pMD2.G) at a ratio of 3:2:1 (w/w/w). After 48 h, the lentivirus‐containing medium was collected and filtered through a 0.45 µm filter. The filtered medium was used to infect HEK‐293T cells in the presence of 8 µg mL^−1^ polybrene (MERCK, Cat: TR‐1003‐G) via spin infection. The HEK‐293T cells with the lentivirus were spinning down at 1000 × *g* at 33 °C for 1.5 h. Following infection, cells were incubated at 37 °C with 5% CO_2_ for the next 6 h, and then the medium was replaced with fresh medium. The antibiotic 5 µg mL^−1^ puromycin was added to the cell culture for 2 weeks for selection. To pick out the single clone cell, ≈100 puromycin‐resistant cells were plated into 96‐well plates, and then the expanded cells were collected for the following experiments.

### Protein Crystallography and Structure Determination

Sec23a/Sec24a (lacking residues 1–340) was concentrated to 15 mg mL^−1^ and mixed with Sec22 at a molar ratio of 1:1.5 for crystallization. Crystals were grown using the hanging drop vapor diffusion method in the buffer containing 10% PEG4K, 0.4 M NaAc, and 0.1 M Tris‐HCl, pH 7.9. For peptide soaking experiments, crystals were incubated with the peptide at 10 mM for 1 h. Next, the crystals were flash‐frozen in liquid nitrogen using a cryoprotectant solution containing the crystallization mother buffer plus 25% ethylene glycol for X‐ray diffraction analysis. Crystallographic data were collected at the BL18U1 and BL19U1 beamlines of the Shanghai Synchrotron Radiation Facility (SSRF). Data scaling and reduction were conducted by HKL3000.^[^ ^81^ ^]^ The structure was solved using molecular replacement method and the structure refinement were both carried out using the Phenix software suite.^[^ ^82^ ^]^ The STING peptides were manually built into the electron density maps using COOT software.^[^ ^83^ ^]^

### Phosphorylation Assay and GST Pull‐Down

The STING phosphorylation assay was performed as previously described.^[^ ^42^ ^]^ 10 mg mL^−1^ GST‐tagged human STING CTT (residue number: 335–379) were phosphorylated by 1 mg mL^−1^ GST‐tagged human TBK1. The reaction mixture was incubated overnight on ice in phosphorylation buffer (20 mM HEPES pH 7.4, 10 mM MgCl_2_, 100 mM NaCl, 5 mM ATP, 0.1 mM Na_3_VO_4_, 5 mM NaF, and 5 mM DTT). The phosphorylated STING was used directly for the pull‐down experiment. The GST pull‐down experiments were conducted as follows: 20 µL of a 50% (v/v) slurry of glutathione Sepharose 4B beads were incubated with phosphorylated GST‐STING protein (0.2 mg) for 1 h at 4 °C. The beads were then washed once with pull‐down buffer (20 mM HEPES‐Na, pH 7.4, 200 mM NaCl, 4 mM DTT, and 0.1% Triton X‐100, 200 µL) by centrifugation at 250 × *g* for 5 min at 4 °C. After washing, the beads were incubated with either COPII protein or IRF3 at a concentration of 1 mg mL^−1^ in pull‐down buffer for 1 h at 4 °C. The beads were washed once with 100 µL of pull‐down buffer, and 20 µL of 5 × protein loading buffer was added to the samples. The samples were boiled at 100 °C for 10 min, centrifuged at 30000 × *g*, and analyzed by SDS‐PAGE.

### Immunofluorescence and Live Cell Fluorescence Imaging

Imaging experiments were performed using mouse STING stable cell lines with an N‐terminal GFP tag, except for the SAVI mutant V155M, which was expressed transiently as a GFP‐tagged protein. The HEK‐293T cells that stably express the mouse STING with a GFP tag were seeded into poly‐L‐lysine‐coated confocal dishes and then the cells were incubated at 37 °C with 5% CO_2_ for 12 h. Next, the cells were treated with 50 µM DMXAA for the indicated time. Subsequently, the cells were fixed with 4% paraformaldehyde (PFA) at room temperature for 10 min, followed by permeabilization with 0.25% Triton X‐100 for 10 min at room temperature. After blocking with 5% bovine serum albumin (BSA, w/v) in PBS for at least 1 h, cells were incubated with primary antibodies against Sec61β, ERGIC53, or GM130 at 4 °C overnight. Following primary antibody incubation, cells were treated with a goat anti‐rabbit IgG (H+L) secondary antibody for 1 h at room temperature. The cell nucleus was stained by Hoechst 33 342 prior to imaging. For live cell imaging, cells were imaged immediately after stimulation at the indicated time. All confocal images were acquired using an Olympus FV3000 confocal microscope (Japan) equipped with a 100 × oil immersion lens.

### Western Blot

Mammalian cells were lysed on ice for 45 min in buffer containing 50 mM HEPES, pH 7.4, 150 mM NaCl, 1 mM EDTA, 1% Triton X‐100, and 1% NP‐40, supplemented with EDTA‐free protease inhibitor cocktail (Roche, Cat: 12352204). Lysates were clarified by centrifugation (20000 × *g*, 10 min, 4 °C), and supernatants were resolved on 4%–20% gradient SDS‐PAGE gels. Proteins were transferred to PVDF membranes (Thermo Fisher, Cat: 88518) and blocked with 5% non‐fat milk for 1 h. Membranes were probed with primary antibodies (following manufacturer‐recommended protocols), washed 3 × with PBST (0.1% Tween‐20 in 1× PBS), and incubated with HRP‐conjugated secondary antibodies (diluted as specified) for 1 h at room temperature. After 3 × PBST washes, protein bands were visualized using enhanced chemiluminescence (ECL) substrate (Thermofisher, Cat: 32106) and imaged.

### Native PAGE Assay

For native protein analysis, cells were lysed in 20 µL of ice‐cold native lysis buffer (50 mM NaCl, 50 mM imidazole, 2 mM 6‐aminohexanoic acid, 1 mM EDTA, pH 7.0) containing 2% digitonin and protease inhibitor cocktail, followed by 45 min incubation on ice. After centrifugation (15000 × *g*, 15 min, 4 °C), the supernatant was combined with 5 × native loading buffer (0.5% Coomassie Brilliant Blue G‐250, 0.66 M 6‐aminohexanoic acid in deionized water). Samples were resolved on an 8% nondenaturing polyacrylamide gel using native electrophoresis buffer at 120 V for 3–4 h with continuous cooling in an ice bath. For protein detection, membranes were processed as previously described for Western blot analysis.

### RT‐qPCR

Gene expression analysis of STING downstream targets was performed using SYBR Green‐based quantitative real‐time PCR. Total RNA was isolated from cells using the RNAprep Pure Cell/Bacteria Kit (TIANGEN, Cat: 4992235) according to the manufacturer's protocol. RNA quality was verified by spectrophotometric analysis (NanoDrop), with all samples demonstrating A260/A280 ratios between 1.9‐2.1, indicating high purity. First‐strand cDNA synthesis was carried out using the FastKing RT Kit (TIANGEN, Cat: 4992224) with genomic DNA removal.

Quantitative PCR reactions were performed in triplicate using the SuperReal PreMix Plus (SYBR Green) kit (TIANGEN, Cat: 4992215) on a QuantStudio 6 Flex Real‐Time PCR System (Applied Biosystems). Thermal cycling conditions consisted of an initial denaturation at 95 °C for 15 min, followed by 40 cycles of 95 °C for 10 s and 60 °C for 30 s. Gene expression levels were normalized to GAPDH as an endogenous control and calculated using the comparative 2^−ΔΔCt^ method. Primer sequences are provided in Table S2 (Supporting Information).

### In Vitro COPII Budding Assay

The preparation of semi‐permeable cells was performed as previously described.^[^ ^18^, ^19^ ^]^ Briefly, 293T‐STING cells at less than 60% confluence were grown in two 15‐cm petri dishes for 48 h. The cells were washed twice with ice‐cold PBS, treated with 2 mL of trypsin, and digested at room temperature for 1–2 min. Digestion was stopped by adding 200 µL of soybean trypsin inhibitor (1 mg mL^−1^). The cells were then resuspended in 10 mL of ice‐cold KHM buffer (20 mM HEPES, pH 7.3, 110 mM potassium acetate, 2 mM magnesium acetate) and centrifuged at 250 × *g* and 4 °C for 3 min to collect the cell pellet. The supernatant was removed, and the cells were resuspended in 6 mL of KHM buffer (50 mM HEPES, pH 7.3, 90 mM potassium acetate). The cells were permeabilized on ice with 10 µg mL^−1^ digitonin for 5 min, followed by the rapid addition of 8 mL of KHM buffer and centrifugation at 250 × *g* and 4 °C for 3 min. The cells were then resuspended in 10 mL of HEPES buffer, incubated on ice for 10 min, and centrifuged at 250 × *g* and 4 °C for 3 min. The pellet was resuspended in 1 mL of KHM·Cl buffer (110 mM potassium chloride, 20 mM HEPES, pH 7.3, 2 mM magnesium chloride) and centrifuged at 10000 × *g* at 4 °C for 15 s. Finally, the cells were resuspended in ≈100 µL KHM·Cl buffer, and the protein concentration was adjusted to 5–8 mg mL^−1^. The prepared semi‐permeable cells were used immediately for the COPII budding assay.

The STING pathway activation reconstitution was modified slightly based on previous COPII vesicle cell‐free reconstitution reports.^[^ ^18^, ^19^ ^]^ The COPII vesicle reconstitution was first conducted and then the 50 µL reaction were conducted with the system containing cell cytosol and semi‐permeabilized cells that stably express STING, both of which were prepared in KHM·Cl buffer supplemented with GTP (0.2 mM), an ATP regeneration system (40 mM creatine phosphate, 0.2 mg mL^−1^ creatine phosphokinase, 1 mM ATP), the protease inhibitor cocktail, and a phosphatase inhibitor. The reaction was incubated at 30 °C for 45 min to reconstitute COPII vesicles. The reaction was then terminated by placing the mixture on ice. A 10 µL aliquot of the reaction mixture was mixed with 25 µL of 2 × protein loading buffer and supplemented with the PBS buffer to a final volume of 50 µL to serve as the total reaction sample. The remaining 40 µL of the reaction mixture was centrifuged at 12000 × *g* for 20 min at 4 °C. The resulting pellet was resuspended in 100 µL of 2 × protein loading buffer and supplemented with PBS buffer to a final volume of 200 µL to serve as the pellet sample. The supernatant containing reconstituted COPII vesicles was transferred to polypropylene centrifuge tubes (Thermo Scientific, Cat: 54 459) and centrifuged at 100000 × *g* for 1 h at 4 °C using a Thermo Scientific TFT 80.2 rotor to collect the vesicle pellet. The vesicle pellet was resuspended in 30 µL of 1× protein loading buffer, while 100 µL of 2 × protein loading buffer was added to the supernatant and supplemented with PBS buffer to a final volume of 200 µL to serve as the supernatant sample. All samples were boiled at 100 °C for 2 min and subsequently analyzed by Western blot.

### IFN‐β Luciferase Reporter System Assay

HEK‐293T cells stably expressing human STING WT or mutants were seeded in 96‐well plates at a density of 2 × 10⁴ cells per well. When the cells reached 60%–70% confluency, they were transiently transfected with pGL3 IFNβ‐luc (50 ng per well) using a lipofectamine‐based transfection reagent. After 18 h of transfection, cells were permeabilized with digitonin and treated with 10 µM cGAMP. Following cGAMP treatment, the medium was replaced with a fresh complete medium, and the cells were returned to the incubator for further culture. 6 h later, Dual‐Glo Luciferase Reagent (Promega) was added in equal volume to the culture medium to lyse the cells completely. Firefly luciferase activity was measured within 2 h using a SpectraMax i3x microplate reader (Molecular Devices) according to the manufacturer's manual.

### Co‐Immunoprecipitation

HEK‐293T cells stably expressing hSTING were seeded into 10 cm cell culture plates, and when the cells reached 60%–70% confluency, Sec24a with a FLAG tag at the C‐terminus was transiently transfected into the cells. After 48 h of transfection, whole cells were collected and lysed to obtain cell lysate, 300 µL of cell lysate was mixed with 30 µL of 50% magnetic bead slurry and incubated at 4 °C for 2 h. After incubation, the magnetic beads were collected using a magnetic rack, DynaMagTM‐2, and were washed once with 100 µL of elution buffer (50 mM HEPES pH 7.4, 150 mM NaCl, 1 mM EDTA, 0.2% Triton X‐100). The bead wash buffer was aspirated, and the sample was boiled at 100 °C for 10 min with protein loading buffer and subsequently analyzed by Western blot.

### Quantification and Statistical Analysis

The data were presented as means ± standard error of the mean (SEM) from at least three biological replicates. Statistical significance of fluorescence intensity, Western blot, and RT‐qPCR results was determined by 2‐tailed unpaired Student's t‐test between two groups or one‐ or two‐way ANOVA between multiple groups. Differences were considered statistically significant at confidence levels of *p < 0.05, **p < 0.01, ***p < 0.001 or ****p < 0.0001. All statistical analyses were performed using GraphPad Prism software (version 8.3 for Windows, GraphPad Software, Inc.). Cellular fluorescence levels from microscopy images and gray values of Western blot were quantified using ImageJ.

## Conflict of Interest

The authors declare no conflict of interest.

## Author Contributions

Y.N., D.C., and J.G. contributed equally to this work. W.M. and A.X. designed the study. Y.N., X.M., and M.Y. performed the X‐ray crystallographic analysis. Y.N., D.C., J.G., J.W., L.G., T.L., and G.H. conducted all other experiments, excluding the structural biology investigations. All authors participated in data analysis, and the original manuscript was written by W.M., Y.N., D.C., and J.G.

## Supporting information



Supporting Information

## Data Availability

The data that support the findings of this study are available from the corresponding author upon reasonable request.
