# Supplementary material for: STING COPII ER Export Trafficking and Signaling Primed by Phosphorylation Switches
Source: Adv Sci (Weinh). 2025 Jul 1;12(36):e03660. doi: 10.1002/advs.202503660 (PMC12463132; doi:10.1002/advs.202503660)
Supplement: Supplementary file 1 — Supporting Information [file ADVS-12-e03660-s001.docx]

Supporting Information

Title：STING COPII ER Export Trafficking and Signaling Primed by a Phosphorylation Switch

Yanan Nan, Dongxiao Cui, Jiajian Guo, Xiaojing Ma, Jiaming Wang, Linyue Guo, Tianyu Li, Mingrui Yang, Guangrui Huang, Anlong Xu^*^, Wenfu Ma^*^

Supporting Display items：Figure S1 to S5, Tables S1 to S2


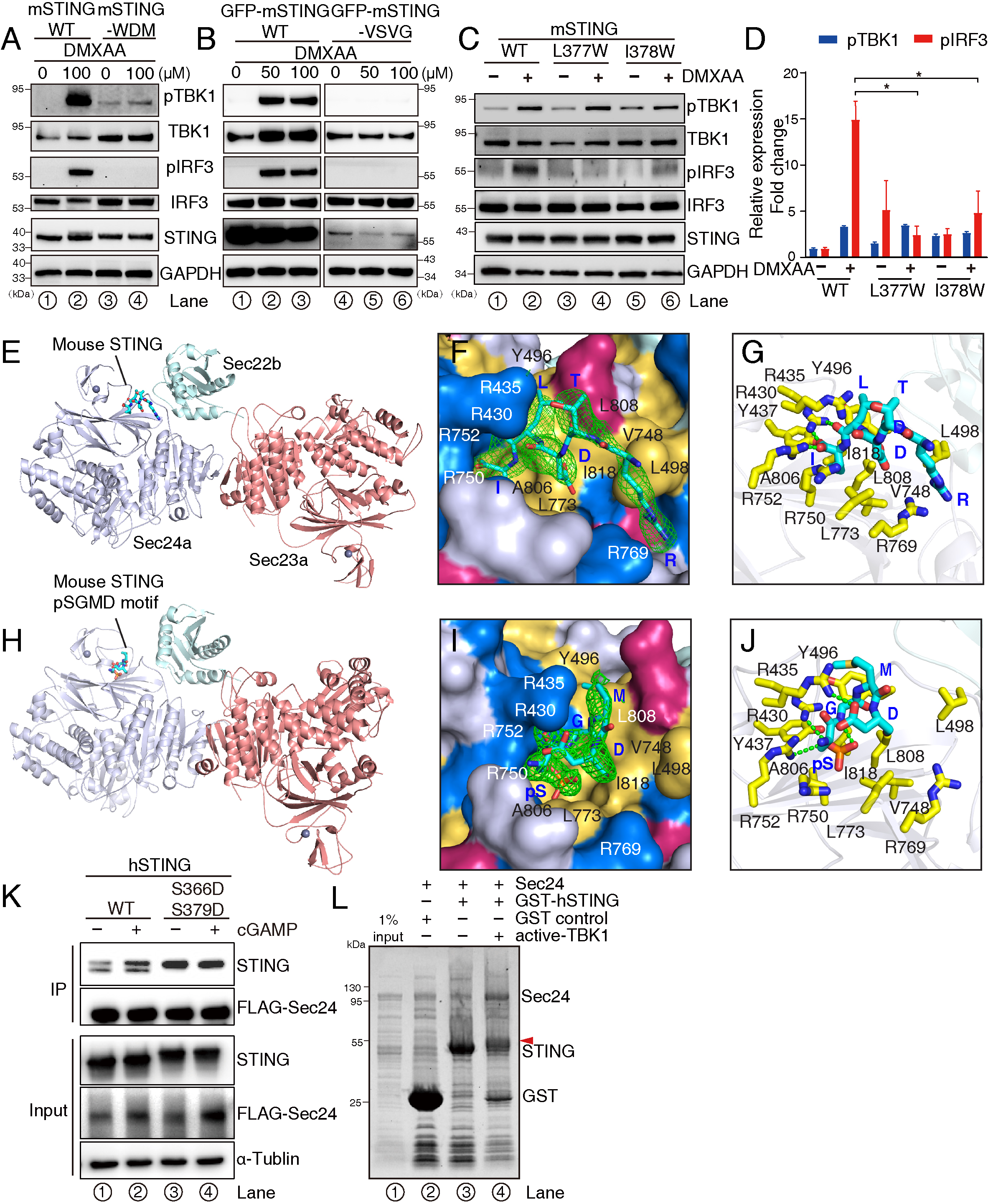


**Figure S1. Structural and functional characterization of the ΦC and pSGMD motifs in mouse STING.** (**A-B**) Western blot analysis of TBK1 activation and IRF3 phosphorylation in HEK-293T cells stably expressing STING-WDM and GFP-STING-VSVG after treatment with DMXAA for 1 h.(**C-D**) Western blot analysis of TBK1 activation and IRF3 phosphorylation in STING mutants L377W and I378W, which disrupt the ΦC motif (C), and (D) shows the quantitative analysis of the data presented in (C). Data is presented as mean ± SEM after analysis of one-way ANOVA, n = 3. **P* < 0.05.(**E-G**) Interaction of the mouse STING ΦC motif with the Sec23a/24a/22b complex (PDB ID: 9UVF), shown in cartoon representation (E), with a close-up view of the interaction (F-G). The difference density map is contoured at 2.0 σ, at a resolution of 3.1 Å (Table S1). (**H-J**) Binding of the mouse pSGMD motif to the Sec23a/24a/22b complex (PDB ID: 9UVG), illustrated in cartoon representation (H), with detailed close-up views (I-J). The difference density map is contoured at 2.0 σ, at a resolution of 2.4 Å (Table S1). The ΦC and pSGMD motifs are depicted in cyan stick representation, with key residues in the B sites shown in yellow. Positively charged residues are colored blue, negatively charged residues red, and hydrophobic residues yellow. (**K**) Interaction between Sec24a and phosphorylated or phosphomimetic STING examined by Co-IP experiment. The FLAG tagged Sec24a was utilized as bait while STING as the target with or without cGAMP stimulation. (**L**) Direct interaction between Sec24a and phosphorylated STING examined by GST pull down assay. The N-terminal GST-fused native or TBK1-mediated phosphorylated STING (residue number: 140-379) were utilized as bait to pull down Sec24a.


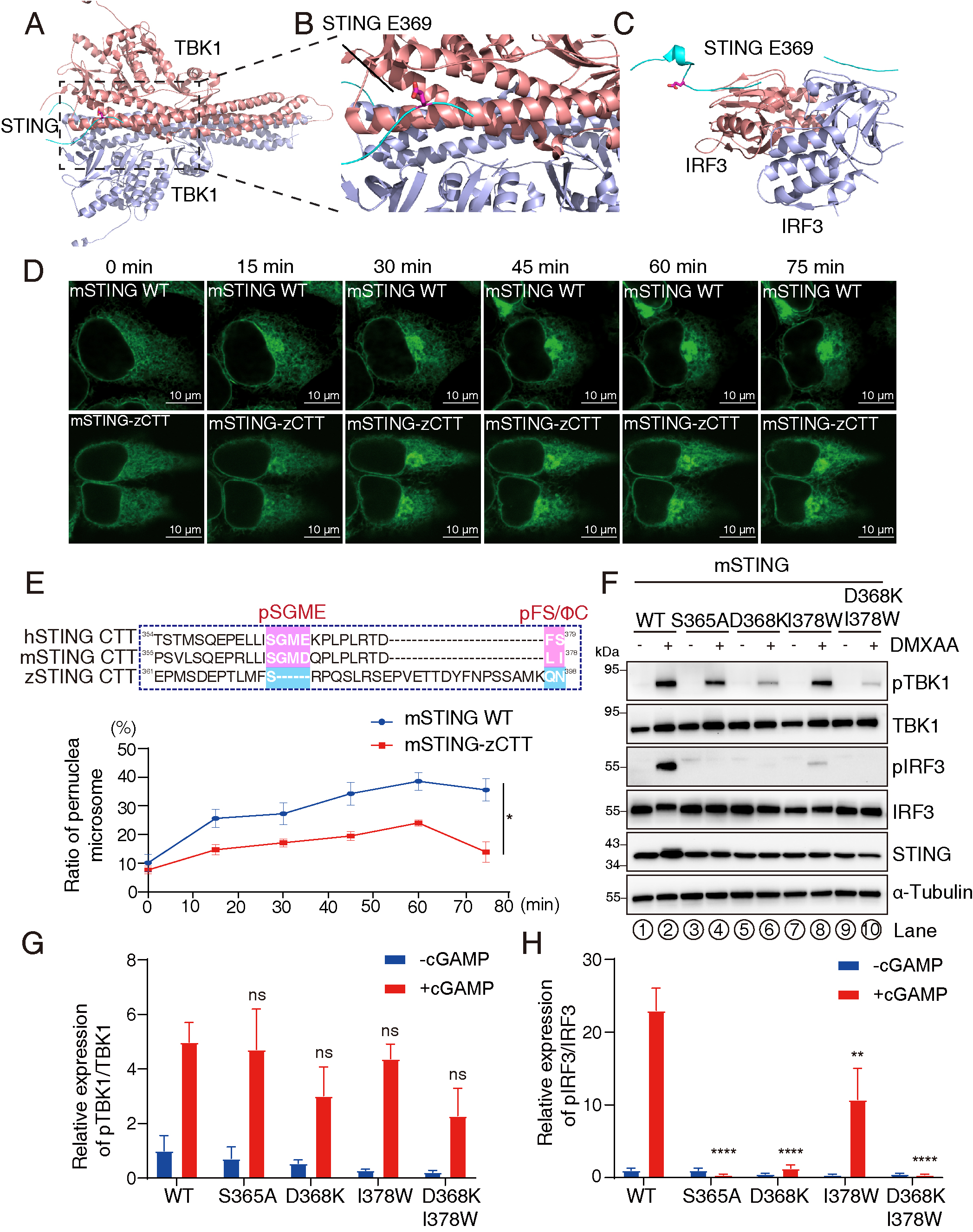


**Figure S2. Structural and functional characterization of the COPII sorting motifs in mouse STING.** (**A-C**) STING E369 is not required for interactions with TBK1 or IRF3. (**D**) Representative confocal images of mouse WT STING and chimeric STING with the zebrafish C-terminal tail (zCTT). (**E**) Quantitative analysis of (**D**) based on the ratio of perinuclear microsome to total STING. Data is presented as mean ± SEM after analysis of two tailed unpaired T test, n = 18. **P* < 0.05. (**F-H**) Western blot analysis of HEK-293T cells stably expressing mouse STING downstream signaling pathways using pSGMD or ΦC-defective STING mutations after treatment with DMXAA for 1 h (F). (G-H) Relative expression analysis of (F). Data is presented as mean ± SEM after analysis of two-way ANOVA analysis, n = 3. ns indicates no significant difference, ***P* < 0.01, *****P* < 0.0001, compared to WT + cGAMP group.


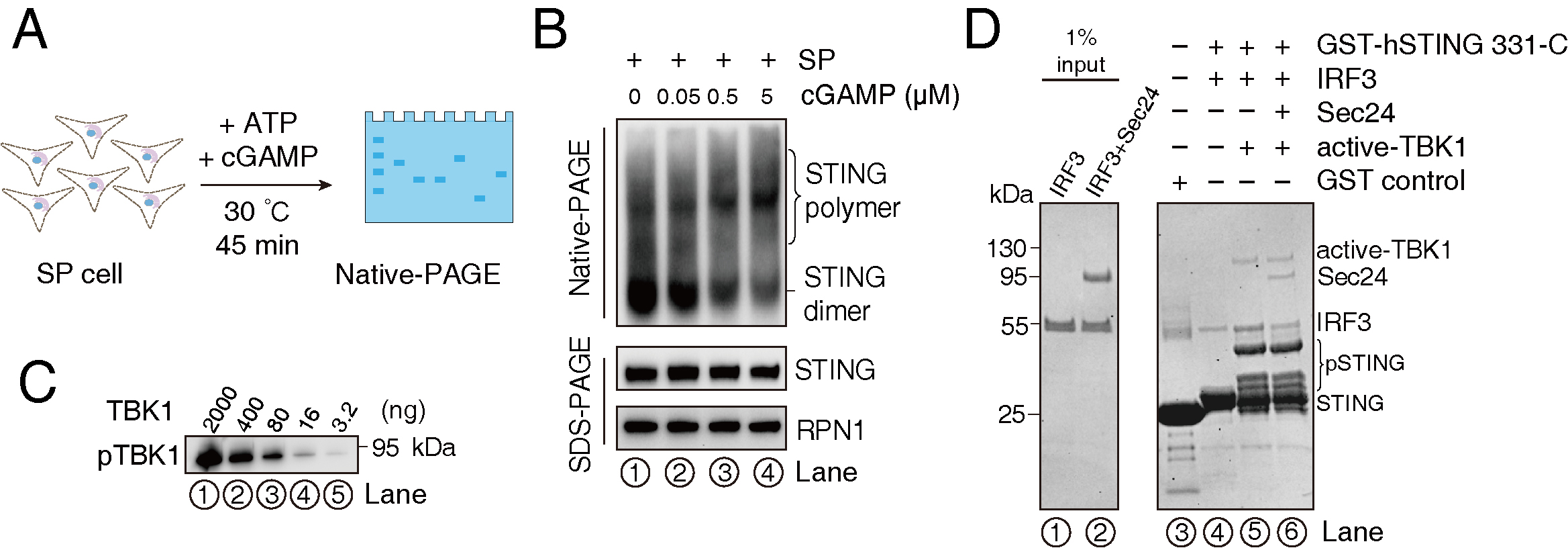


**Figure S3. cGAMP-induced oligomerization of STING in the SP membrane and Sec24-mediated attenuation of IRF3 binding to phosphorylated STING.** (**A**) Schematic illustrating the analysis of STING behavior using native SDS-PAGE. (**B**) STING oligomerization in response to increasing concentrations of cGAMP. (**C**) Western blot analysis of active recombinant TBK1 purified from insect cells. (**D**) GST pull down assays demonstrate the competitive interaction between IRF3 and Sec24 for binding to phosphorylated STING.


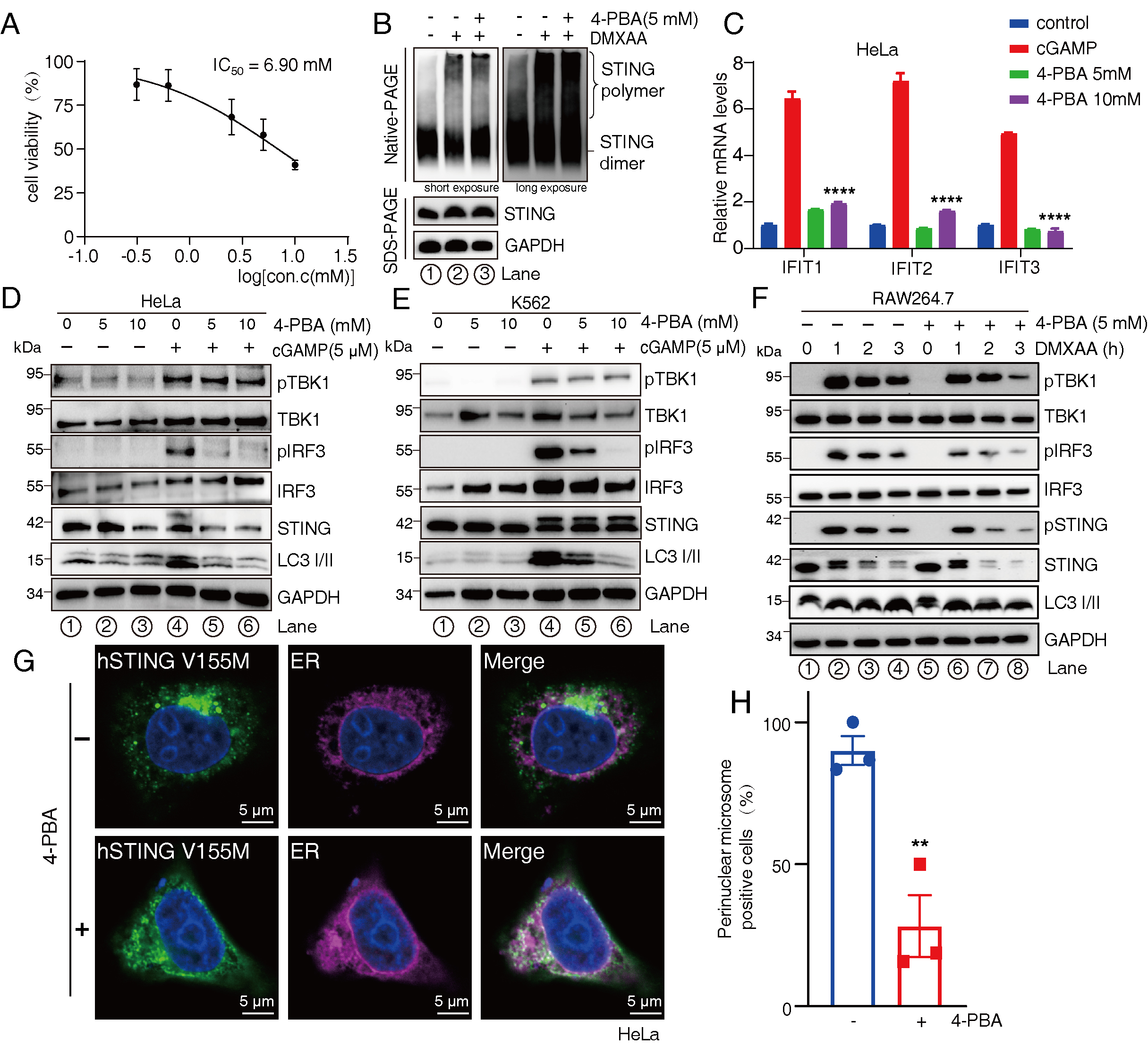


**Figure S4. 4-PBA inhibits STING signaling by reducing its ER export.** (**A**) IC_50_ curve of 4-PBA in HEK-293T cells. (**B**) 4-PBA does not inhibit DMXAA-triggered STING oligomerization in HEK-293T cells. (**C**) *IFIT1-3* gene expression assessed by RT-qPCR following treatment with 4-PBA and cGAMP. Data is presented as mean ± SEM after analysis of one-way ANOVA, n = 3. *****P* < 0.0001. (**D-E**) Western blot analysis of STING downstream signaling in HeLa or K562 cells (human STING) following treatment with 4-PBA and cGAMP. (F) Western blot analysis of RAW264.7 cells endogenously expressing STING were stimulated with 50 µM DMXAA for the indicated time after pre-treatment with 5 mM 4-PBA for 2 h. (**G**) Representative confocal images showing the reduction in puncta ratio induced by SAVI-related STING mutation with 4-PBA treatment. (**H**) Quantitative analysis of percentage of STING perinuclear microsome positive cells for (G) from 3 independent experiments (66 cells analyzed). Data is presented as mean ± SEM after analysis of two tailed unpaired T test, n = 3 ***P* < 0.01.


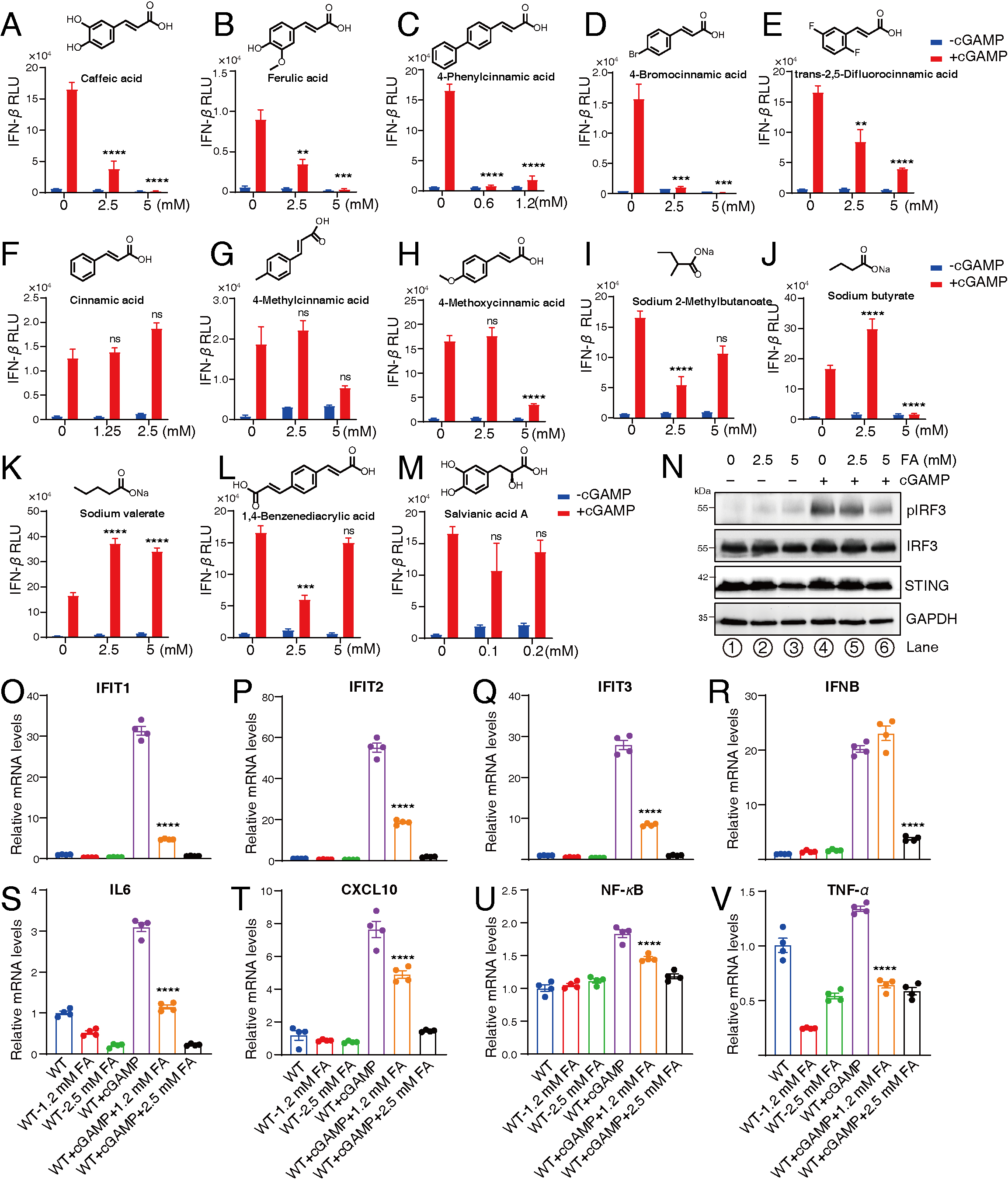


**Figure S5. Identification of 4-PBA-like compounds that inhibit STING signaling using an IFN-β luciferase reporter system.** (**A-M**) Screening of ten 4-PBA-like compounds and three short-chain fatty acids, with five hit compounds shown in (A-E). Data is presented as mean ± SEM after analysis of one-way ANOVA analysis, n = 3. *****P* < 0.0001, ns indicates no significant difference, compared to 0 mM + cGAMP group. (**N)** Western blot analysis of HEK-293T cells stably expressing human STING were stimulated with 5µM cGAMP for 1 h after pre-treatment with indicated concentration of ferulic acid (FA) for 2 h. **(O-V**) STING downstream signaling analysis following treatment with ferulic acid (FA), assessed by RT-qPCR. Data is presented as mean ± SEM after analysis of one-way ANOVA analysis, n = 4. *****P* < 0.0001, compared to WT + cGAMP group.

**Table S1. Data collection and refinement statistics of complex crystal structure composing of Sec23a/24a/22b and STING COPII-sorting motifs**

| Crystal  Motif  PDB ID  Space group:  Copies per ASU  Cell parameters a, b, c (Å)  α, β, γ (°) | COPII-hSTING  pSGME:LLIpSGMEK  9UVD  C2  1  149.1,95.9,130.1  90,90.2,90 | COPII-hSTING  pFS: RTDFpS  9UVE  C2  1  149.2,98.1,130.1  90.0,90.3,90.0 | COPII-mSTING  pSGMD:LLIpSGMDQ  9UVG  C2  1  76.2,76.2,55.3  90.0,90.0,90.0 | COPII-mSTING  ΦC:PLRTDLI  9UVF  C2  1  70.3,94.1,134.3  90.0,93.3,90.0 |
| --- | --- | --- | --- | --- |
| **Data processing**  Resolution (Å)  R_merge_ (%)^a^  I/σ  Completeness (%)  Redundancy  CC(1/2) | 50-2.76  10.8(113.5)  9.1(1.3)  99.7(99.9)  3.8(3.9)  0.99(0.56) | 50-2.4  11.7(101.1)  9.2(1.0)  99.9(76.0)  4.6(3.1)  0.99(0.71) | 50-2.54  8.6 (89.5)  7.3(1.1)  99.7(60.1)  4.3(2.4)  0.99(0.89) | 50-3.1  11.8(110.2)  8.8(1.1)  98.6(97.4)  4.6(3.6)  0.99(0.80) |
| **Refinement statistics**  Data range (Å)  Reflections  Nonhydrogen atoms  Water molecules  R.m.s. ∆bonds (Å)^b^  R.m.s. ∆ angles (°)^b^  R-factor (%)^c^  Rfree (%)^c, d^ | 50-2.76  47471  12491  86  0.004  0.65  23.7  26.5 | 50-2.4  67680  12513  99  0.003  0.5  23.6  25.1 | 50-2.54  53284  12480  95  0.004  0.7  20.6  22.4 | 50-3.1  31307  12450  31  0.003  0.6  25.1  27.6 |

*Highest resolution shell is shown in parenthesis.

^a^ R_merge_ = 100 x ∑_h_∑_i_ | *I*_i_(h) - <*I*(h)> | / ∑_h_<*I*(h)> , where *I*_i_(h) is the *i*th measurement and <*I*(h)> is the weighted mean of all measurement of *I*(h)

for Miller indices h.

^b^ Root-mean-squared deviation (r.m.s. ∆) from target geometries.

^c^ R-factor = 100 x ∑|F_P_ – F_P(calc)_|/∑ F_P_.

^d^ R_free_ was calculated with 5% of the data

| **Gene Name** | **Forward (5’ to 3’)** | **Reverse (5’ to 3’)** |
| --- | --- | --- |
| IFIT1 | TTGATGACGATGAAATGCCTGA | CAGGTCACCAGACTCCTCAC |
| IFIT2 | AAGCACCTCAAAGGGCAAAAC | TCGGCCCATGTGATAGTAGAC |
| IFIT3 | TCAGAAGTCTAGTCACTTGGGG | ACACCTTCGCCCTTTCATTTC |
| IFN-β | CATTACCTGAAGGCCAAGGA | CAGCATCTGCTGGTTGAAGA |
| CXCL10 | TCCACGTGTTGAGATCATTGCTAC | CTGTGTGGTCCATCCTTGGAA |
| IL6 | GTCCTGATCCAGTTCCTGCAG | GTTCTGTGCCCAGTGGACAGG |
| TNF-α | TCTTCTGCCTGCTGCACTTTG | ACCTTGGTCTGGTAGGAGACG |
| NF-κB | GCAGATGGCCCATACCTTCA | TCCCACATAGTTGCAGATTTTGAC |
| GAPDH | GAGTCAACGGATTTGGTCGT | GACAAGCTTCCCGTTCTCAG |

**Table S2. Primer sequence of RT-qPCR**
